# Phenotypic Selection in Ornamental Breeding: It's Better to Have the BLUPs Than to Have the BLUEs

**DOI:** 10.3389/fpls.2018.01511

**Published:** 2018-11-05

**Authors:** Heike Molenaar, Robert Boehm, Hans-Peter Piepho

**Affiliations:** ^1^Biostatistics Unit, Institute of Crop Science, University of Hohenheim, Stuttgart, Germany; ^2^Klemm + Sohn GmbH & Co. KG, Stuttgart, Germany

**Keywords:** BLUP, BLUE, two-phase design, phenotypic selection, family-index selection, individual selection, ornamental breeding

## Abstract

Plant breeders always face the challenge to select the best individuals. Selection methods are required that maximize selection gain based on available data. When several crosses have been made, the BLUP procedure achieves this by combining phenotypic data with information on pedigree relationships via an index, known as family-index selection. The index, estimated based on the intra-class correlation coefficient, exploits the relationship among individuals within a family relative to other families in the population. An intra-class correlation coefficient of one indicates that the individual performance can be fully explained based on the family background, whereas an intra-class correlation coefficient of zero indicates the performance of individuals is independent of the family background. In the case the intra-class correlation coefficient is one, family-index selection is considered. In the case the intra-class correlation coefficient is zero, individual selection is considered. The main difference between individual and family-index selection lies in the adjustment in estimating the individual's effect depending on the intra-class correlation coefficient afforded by the latter. Two examples serve to illustrate the application of the BLUP method. The efficiency of individual and family-index selection was evaluated in terms of the heritability obtained from linear mixed models implementing the selection methods by suitably defining the treatment factor as the sum of individual and family effect. Family-index selection was found to be at least as efficient as individual selection in *Dianthus caryophyllus* L., except for flower size in standard carnation and vase life in mini carnation for which traits family-index selection outperformed individual selection. Family-index selection was superior to individual selection in *Pelargonium zonale* in cases when the heritability was low. Hence, the pedigree-based BLUP procedure can enhance selection efficiency in production-related traits in *P. zonale* or shelf-life related in *D. caryophyllus* L.

## Introduction

For decades “Best Linear Unbiased Prediction” (BLUP) has been the standard selection method in animal breeding (Henderson, 1950), where the breeding values of sires are estimated based on progeny performance to select superior genotypes and to breed superior families (Robinson, 1991). More recently, this method has been used in commodity crops (Piepho et al., 2008) and has also been applied in several clonally propagated species such as sweet potato (Borges et al., 2010), acai berry (Teixeira et al., 2012), potato (Slater et al., 2014; Ticona-Benavente and da Silva Filho, 2015), sugar cane (Barbosa et al., 2005; Zeni Neto et al., 2013), and passion fruit (Santos et al., 2015). Currently, the pedigree-based BLUP method is replaced by genomic prediction in many species (Gianola et al., 2018). In comparison to the pedigree-based BLUP, genomic prediction uses a marker-based matrix of genomic pair-wise similarities known as “genomic relationship matrix” (Van Raden, 2008; Legarra, 2016; Wang et al., 2017). Furthermore, the pedigree-based genetic variance-covariance matrix is replaced by the genomic variance (Lehermeier et al., 2017). However, marker data are severely limited in ornamental breeding programs. Thus, the pedigree-based BLUP method proposed in the present study is currently the most promising selection method to use when no marker-data is available. By this method, useful information can be obtained as to whether the trait is dependent or independent of the family background. This information is vital for selecting individuals for genotyping, because the goal of creating diversity panels is to represent the entire genetic diversity of parental populations, i.e., individuals should be selected with similar biotic or abiotic adaptation or photoperiod requirements (Singh and Singh, 2015, p. 220).

Before BLUP-based selection, selection in crop breeding was based on either simple arithmetic means or “Best Linear Unbiased Estimation” (BLUE) of genotypes, which are calculated in a mixed model context based on fixed genotype effects (Piepho et al., 2008). By contrast, BLUPs are obtained by defining the genotypes as random effects. By convention, “estimation” refers to fixed effects and “prediction” refers to random effects, even though both refer to estimators of effects in a linear mixed model. The first three letters of the acronyms BLUE and BLUP stand for *Best*, meaning they have the lowest variance, *Linear*, meaning they are linear functions of the data, and *Unbiased*. In case of BLUE, unbiased means the expected value of a mean estimate for an individual equals its true value. This is a conditional mean. By contrast, in case of BLUP the expected mean over all individuals is equal to the expected mean over all true effects. This is a marginal mean. The BLUP-based selection method predicts genetic effects more accurately than the BLUE-based method (Copas, 1983; Robinson, 1991). The gain in accuracy compared to BLUE-based selection results partly from the shrinkage property (Piepho et al., 2008), i.e., above average individual means will be shrunken downwards toward the overall mean, whereas below average individual means will be shrunken upwards toward the overall mean. The degree of shrinkage also depends on environmental variation (Hill and Rosenberger, 1985). This shrinkage property anticipates the regression to the mean observed in the selected progeny and is advantageous for selection decisions because individuals with extreme high or low performances are adjusted, which is consistent with the need for caution in making selection decisions on such extremes (Hill and Rosenberger, 1985). A further source of gain in accuracy is the facility to borrow strength from individuals in the same family (Piepho et al., 2008; Bernardo, 2010).

Currently, selection in ornamentals (Boxriker et al., 2017a,b; Molenaar et al., 2017) is based on individual performance, which is known to be a poor strategy when heritability is low. Alternatively, response to selection could be improved by considering family information. The simplest form of selection considering pedigree information is family selection, where selection is based on family means (Lynch and Walsh, 2013). A refinement of family selection is family-index selection (Lush, 1947), which incorporates the individual mean with the family mean (Lynch and Walsh, 2013). Generally, the exploitation of family information can provide greater accuracy and larger response to selection. In particular, index selection has an expected response at least as large as individual selection and even higher responses when significant effects of environmental conditions and replication of families over environments exist (Lynch and Walsh, 2013).

To our knowledge, the BLUP-based selection method has been used only in a few ornamental species so far. Huang et al. (1995) used the BLUP-based selection method to investigate the long-term genetic improvement in 16 generations of gerbera cut-flowers. In the past, software restrictions precluded directly obtaining BLUPs from the so-called “Mixed Model Equations” (MME; Henderson, 1950). Instead, facing computational constraints, Huang et al. (1995) obtained BLUPs by an indirect approach of successive averaging of genotypic effects (Misztal and Gianola, 1987) and the variance components were estimated by the derivative-free restricted maximum likelihood (Graser et al., 1987). Fogaça et al. (2012) used BLUP in daylily breeding and found that higher selection gain is expected from family selection rather than from individual selection. The BLUPs of individuals were obtained by the use of SELEGEN-REML/BLUP software (Resende, 2016).

The present work aims to demonstrate the application of BLUP-based selection in *Pelargonium zonale* and *Dianthus caryophyllus* L., two species which have the highest economic importance in the floricultural industry, and to further demonstrate the enhancement of breeding efficiency. We will briefly review the theoretical underpinnings of BLUP and individual and family-index selection. Then we compare the efficiency of strategies underlying the individual and family-index selection in terms of heritability.

## Materials and methods

### Theoretical underpinnings of BLUE and BLUP

The context of BLUE and BLUP is the standard linear mixed model (LMM; Robinson, 1991; Piepho, 1994),

y = Xβ + Zu + e,

where ***y*** is a vector of *n* observations, **β** is a vector of fixed effects, ***X*** and ***Z*** are design matrices associated with the fixed and random effects, ***u***, the vector of random effects assumed to be distributed according to ***u*** ~ MVN(**0**, ***G***) where **0** is a null vector and ***G*** is the variance-covariance matrix of the random effects, and ***e*** is the vector of residual errors assumed to be distributed as ***e*** ~ MVN(**0**, ***R***) with ***R*** the variance-covariance matrix of the residual errors. The distribution of observed data is assumed to be ***y*** ~ MVN**(*X***β**, *V*)**, where ***V*** accounts for random effects and residual error by ***V*** = ***ZGZ***^**T**^ + ***R***.

The fixed effects (BLUEs) are estimated by $\hat{\beta }\;={\left(X^{T}{\hat{V}}^{-1}X\right)}^{-1}X^{T}{\hat{V}}^{-1}y$, where as the random effects (BLUPs) are predicted by $\hat{u}$ = $\hat{G}Z^{T}{\hat{V}}^{-1}\left(y\;-\;X\hat{\beta }\right)$.

The BLUE and BLUP of **β** and ***u***, respectively, are best computed by solving the MME, given by (Henderson, 1950; Searle et al., 1992),

$$\left[\begin{array}{c} \;X^{T}R^{-1}X\;X^{T}R^{-1}Z\\ Z^{T}R^{-1}X\;Z^{T}R^{-1}Z\;+\;G^{-1} \end{array}\right]\left[\begin{array}{c} \hat{\beta }\\ \hat{u} \end{array}\right]=\;\left[\begin{array}{c} X^{T}R^{-1}y\\ Z^{T}R^{-1}y \end{array}\right]\cdot$$

where ***G***^−1^ and ***R***^−1^ are the inverses of ***G*** and ***R***, respectively.

When ***G***^−1^ tends to a zero matrix, which happens when variances in ***G*** become very large, the random effect estimates behave essentially like fixed effect estimates because the MME tend to

$$\left[\begin{array}{c} X^{T}R^{-1}X\;X^{T}R^{-1}Z\\ Z^{T}R^{-1}X\;Z^{T}R^{-1}Z \end{array}\right]\left[\begin{array}{c} \hat{\beta }\\ \hat{u} \end{array}\right]=\;\left[\begin{array}{c} X^{T}R^{-1}y\\ Z^{T}R^{-1}y \end{array}\right].$$

If furthermore the residual errors are independent with homogenous variance, i.e., ***R***^−1^ = σ^−2^***I***, with σ^−2^ the inverse residual error variance and ***I*** an identity matrix, the MME turn into the ordinary least squares equations (Robinson, 1991),

$$\left[\begin{array}{c} \;X^{T}X\;X^{T}Z\\ \;Z^{T}X\;Z^{T}Z \end{array}\right]\left[\begin{array}{c} \hat{\beta }\\ \hat{u} \end{array}\right]=\;\left[\begin{array}{c} \;X^{T}y\\ \;Z^{T}y \end{array}\right].$$

### Family-index selection

**Figure 1:**
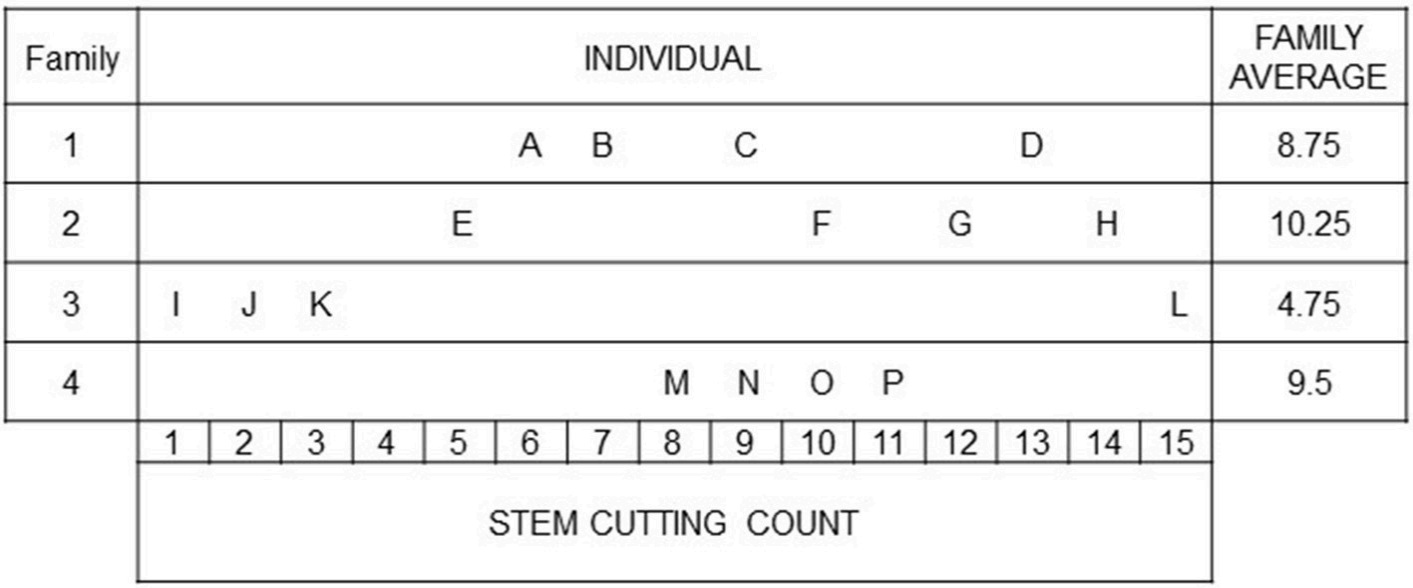
A motivating example to illustrate the individual and family-index selection modified according to (Lush, 1947, pp. 242–244): Stem cutting counts of individuals from four families. The breeder is faced with the choice, e.g., between individuals “O” and “F,” with the same SCC but coming from differently performing families.

The basic idea of family-index selection is to obtain an index that accounts for the resemblance among individuals within a family relative to other families in the population (Lush, 1947). To depict Lush's idea, we give a selection problem in the context of ornamental breeding (modified according to Lush, 1947, pp. 242–244): Four families are considered to select the best performing individuals with respect to stem cutting count (SCC; Figure 1). Different strategies could be taken for selection. Individuals could be selected independently of the performance of their sibs (highest SCC). This method is known as individual selection. An alternative is for the breeder to select a complete family on the basis of family means (family selection). In the example, “Family 2” would be selected showing the highest SCC performance. Combining these two selection methods by considering both individual performances and family means in an index (family-index selection), the breeder would select “F” and “P” rather than “D” and “L.” Independently of the selection method, individuals “G” and “H” will always be selected, because the family average can be high only when more than a substantial proportion of the individuals in a family are above the general population mean (Lush, 1947). Furthermore, it will almost never happen that all individuals of the superior family are superior to all members of other families (Lush, 1947). The main difference between individual, family and family-index selection consists in what is done with good individuals from mediocre families (like “D” and “P”) and with intermediate individuals (like “F”) or poor individuals (like “E”) from better performing families (Lush, 1947), which is illustrated in the following with a particular emphasis on the partition of variance.

Continuing with the motivating example, it is assumed that for each individual “A” to “P” two observations are available and the design was completely randomized. Selection can based on the LMM

$$y\;=\;1_{n}\mu \;+\;Z_{g}g\;+\;e,$$

where ***y*** is the (*n* × 1) vector of SCC observations, **1**_*n*_μ is the (*n* × 1) vector of ones allocating the general population mean to all observations, ***g*** is the (*s* × 1) vector of random genetic strain effects and distributed as N$\left(\text{0},\;\sigma_{g}^{2}I\right)$ with the genetic strain variance $\sigma_{g}^{2}$ and ***I*** the (*s* × *s*) identity matrix, ***Z***_*g*_ is the (*n* × *s*) design matrix of random strain effects relating observations to strains and the random (*n* × 1) vector ***e*** distributed $N\left(0,\;\sigma_{e}^{2}I\right)$ with the non-genetic $\sigma_{e}^{2}$ variance and ***I*** the (*n* × *n*) identity matrix. Given this baseline model, the phenotypic variance is $V\;=\;Z_{g}GZ_{g}^{T}\;+\;R$, where $G\;=\;\sigma_{g}^{2}I_{s\;\times \;s}$ and $R\;=\;\sigma_{e}^{2}I_{n\;\times \;n}$.

To account for the *simple* nested family structure (Piepho and Williams, 2006), i.e., for families and individuals that can be grouped by family, the genetic effect of the baseline LMM is partitioned as ***g*** = ***Z***_*f*_***f*** + ***m***, so that the LMM becomes

$$y\;=\;1_{n}\mu \;+\;Z_{\text{g}}Z_{\text{f}}f\text{ + }Z_{\text{g}}m\text{ + }e,$$

where ***f*** is the (*w* × 1) vector of random family effects assumed to be N$\left(\text{0},\;\sigma_{f}^{2}I\right)$, ***Z***_*f*_ is the (*s* × *w*) design matrix of the random family effects, ***m*** is the (*s* × 1) vector of random effects of individuals nested within family effects assumed to be N$\left(\text{0},\;\sigma_{s}^{2}I\right)$, and the residual term ***e*** is defined as in the baseline model. The resemblance among individuals of each family is given by the intra-class correlation coefficient, *t*,

$t=\;\frac{\sigma_{f}^{2}}{\sigma_{g}^{2}}$, where the total genetic variance is $\sigma_{g}^{2}$ = $\sigma_{f}^{2}\;+\sigma_{s}^{2}$.

On account of the intra-class correlation coefficient, for individuals in the same family the zeros on the off-diagonals of the variance-covariance matrix ***G*** under the baseline model are replaced with the family variance, resulting in a block diagonal ***G*** matrix with blocks corresponding to families. This structure is also known as the compound symmetry (CS) variance-covariance structure. For a single family with four individuals, the block on the diagonal of ***G*** is

$$\sigma_{g}^{2}I_{4\;\times \;4}=\sigma_{g}^{2}\left(\begin{array}{cccc} 1 & t & t & t\\ t & 1 & t & t\\ t & t & 1 & t\\ t & t & t & 1 \end{array}\right)=\left(\begin{array}{cccc} \sigma_{g}^{2} & \sigma_{f}^{2} & \sigma_{f}^{2} & \sigma_{f}^{2}\\ \sigma_{f}^{2} & \sigma_{g}^{2} & \sigma_{f}^{2} & \sigma_{f}^{2}\\ \sigma_{f}^{2} & \sigma_{f}^{2} & \sigma_{g}^{2} & \sigma_{f}^{2}\\ \sigma_{f}^{2} & \sigma_{f}^{2} & \sigma_{f}^{2} & \sigma_{g}^{2} \end{array}\right).$$

The variance-covariance structure ***R*** remains unchanged. The motivating example assumes equal family sizes, which is an idealized condition. Unequal family sizes can be accounted for in BLUP (Appendix Presentation 1 in Supplementary Material).

#### Individual selection

Under a CS variance-covariance structure of ***G***, the off-diagonal elements describe the similarities of individuals within families. If the intra-class correlation coefficient, *t*, tends to one, the one limiting case, all individuals within the same family show about the same performance of a trait. In contrast, if the intra-class correlation coefficient, *t*, tends to zero, the other limiting, the performance of individuals is independent of the family background, which is exploited by individual selection.

### Efficiency of selection methods

As shown, the degree of resemblance between individuals grouped by family can be measured by the genetic variance. The broad-sense heritability is given by

$$H^{2}\;=\;\frac{\sigma_{g}^{2}}{\sigma_{p}^{2}},$$

(Bernardo, 2010, p. 135)

where $\sigma_{g}^{2}$ is the genetic variance, and $\sigma_{p}^{2}$ the phenotypic variance. This broad-sense heritability is sometimes also termed as *repeatability* (Piepho and Möhring, 2007) and is used to evaluate trials; the better a trial, the higher *H*^2^. Similarly, *H*^2^ can be used to determine the best breeding method (Falconer and Mackay, 1996). However, the estimation of *H*^2^ using standard equations such as the one given above underlies strong assumptions: balanced data, non-correlated and homoscedastic genetic effects. If one of these assumptions is violated, there will not be a simple linear relationship between response to selection and selection differential, i.e., the correlation between phenotypic value and response to selection differs between genotypes (Piepho and Möhring, 2007). Different approaches (Holland et al., 2003; Cullis et al., 2006; Oakey et al., 2006; Piepho and Möhring, 2007) have been proposed for situations in which these standard assumptions are invalid.

#### Random and independent genotypes

Cullis et al. (2006) proposed to estimate the heritability when the genotypes are taken as random effects by

$$H^{2}=\frac{\sigma_{g}^{2}}{\sigma_{p}^{2}}$$

where $\bar{v}$ is the mean variance of a difference of two BLUPs and $\sigma_{g}^{2}$ is the genotypic variance. The heritability $H_{C\;}^{2}$ accounts for unbalanced data, but still genotypic effects are assumed to be independent, which would be true, if no relationship among individuals existed. However, when selection is exercised on individuals from different families, resemblance between individuals within families is present and alternative methods should be used (Oakey et al., 2006; Piepho and Möhring, 2007). The most flexible option to account for any modeled variance-covariance structure is to simulate the heritability on an entry-mean basis directly as the squared correlation of ***g*** and $\hat{g}$ given by

$$r^{2}=Q^{-1}\sum_{q\;=1}^{Q}r_{q}^{2},$$

where *Q* is the total number of simulation runs, $r_{q}^{2}$ the sample correlation of the genotypic effects, *g*_*i*_, and the BLUPs, ĝ_*i*_ (Piepho and Möhring, 2007). In cases of independent genotypic effects and a balanced design, the expected squared correlation between true and predicted genotypic effects is approximately

$$\text{E}\left(r^{2}\right)\approx {\left(\frac{\text{cov }\left(g_{i},\;{\hat{g}}_{i}\right)}{\sqrt{\text{ var }\left(g_{i}\right)\text{ var}\left({\hat{g}}_{i}\right)}}\right)}^{2},$$

where $\hat{g\;}\;=\text{ BLUP}\left(g\right)$.

The Monte Carlo standard error of the simulated heritability can be defined as

$$s.e.(r^{2})\;=\;\sqrt{\frac{s_{r^{2}}^{2}}{Q}},$$

where $s_{r^{2}}^{2}$ is defined as

$$s_{r^{2}}^{2}=\;\frac{\sum_{q\;=\;1}^{Q}{\left(r_{q}^{2}-{\bar{r}}^{2}\right)}^{2}}{Q-1}.$$

### Selection based on BLUPs

The use of BLUP requires the assumption of normality (Robinson, 1991), which can be graphically checked by Q-Q plots. In Q-Q plots the standardized BLUPs (Searle et al., 1992, pp. 286-287) were plotted against the normal scores in Q-Q plots. The standardized BLUPs were defined as $\frac{{ĝ}_{i}}{\sqrt{\text{Var}\left[{ĝ}_{i}\right]}}$, where ĝ_*i*_ is the *i*-th estimated genotypic BLUP and var[ĝ_*i*_] is the unconditional variance.

### Application of family-index selection

In the next two sections, the application of family-index selection is illustrated by two examples in ornamental breeding. The general approach to implement the family-index selection by a LMM to select individuals across families is to define the treatment effect as the sum of the family effect and individual within family effect (FM + FM·ENTRY). Both effects are modeled as random and the genetic covariance, between individuals within a family is equal to the variance of the family effect, i.e., var(FM), whereas the covariance of individuals from different families is zero. To implement the model in a way that facilitates estimation of the genotypic value of individuals, we drop the family main effect FM and impose a CS variance-covariance structure on the FM·ENTRY effect for individuals in the same family. This implementation is equivalent to the model with independent effects FM and FM·ENTRY, but is more convenient for predicting the family-index, which may be obtained directly as the BLUP of the effect FM·ENTRY under the CS model (Piepho and Williams, 2006). For the implementation of individual selection by a LMM, only the independent term FM·ENTRY is considered. In sections Determining the best selection method by *H*^2^ and Determining the best selection method by *H*^2^, in which models are derived to simulate *H*^2^ for evaluation of selection methods, it will be explicitly mentioned again which terms are crucial for the implementation of either individual or family-index selection.

### Phenotypic selection in ornamentals: the example of production-related traits in *P. zonale* breeding

Conventionally in the *P. zonale* breeding program of Selecta One (Stuttgart-Mühlhausen, Germany), seeds from crosses made in the first year are sown in the second year. Seedlings are selected with a focus on traits such as early flowering or petal color to reduce the population size for later tests (Figure 1 in Molenaar et al., 2017). Each selected individual is cloned (multiplied by cutting propagation) to enable replicated field trials in the third year focusing on color and flower longevity under field conditions for example. Finally, in the fourth year, candidate varieties are screened for production-related traits.

Due to recent advances in knowledge of the genetics of production-related traits (Molenaar et al., 2017), the assessment of production-related traits in 500 *P. zonale* strains has been shifted from the fourth to the second year in a new experiment, because of the great economic relevance of those traits in the breeding program (Molenaar et al., 2017). However, due to lack of time in the second year for clonal reproduction, this shift results in phenotyping of single plants for production-related traits in year two.

#### Plant material

In 2014, twelve reciprocal crosses were made between ten heterozygous elite *P. zonale* strains to obtain six families segregating in the F_1_ already (Table 1). Families were unrelated by pedigree, except for Families 3, 4, and 5, which all had the parent “E” in common and Families 2 and 5, which had parent “d” in common; individuals across these families were half-sibs (Table 1). The parental strains showed either a superior performance in production-related traits (indicated by capital letter), such as SCC or root formation (RF), or in quality traits, such as petal or leaf color. Between 10 and 113 individuals were obtained per family, amounting to 500 individuals across families.

**Table 1:** Parentage and size of the six *P. zonale* families evaluated in this study.

**Family**	**Number of individuals in each family**	**Number of individuals in each reciprocal**	**Parental genotypes**^†^		
			**Paternal**		**Maternal**
1	113	63	(A	×	b)
		50	(b	×	A)
2	51	3	(C	×	d)
		48	(d	×	C)
3	112	49	(E	×	f)
		63	(f	×	E)
4	60	26	(E	×	g)
		36	(g	×	E)
5	101	8	(E	×	d)
		91	(d	×	E)
6	63	43	(I	×	j)
		20	(j	×	I)
*K_*Total*_*	500				

† *Uppercase letters indicate a superiority in production-related traits of the parental genotype*.

#### Two-phase experimental design in *P. zonale* breeding

**Figure 2:**
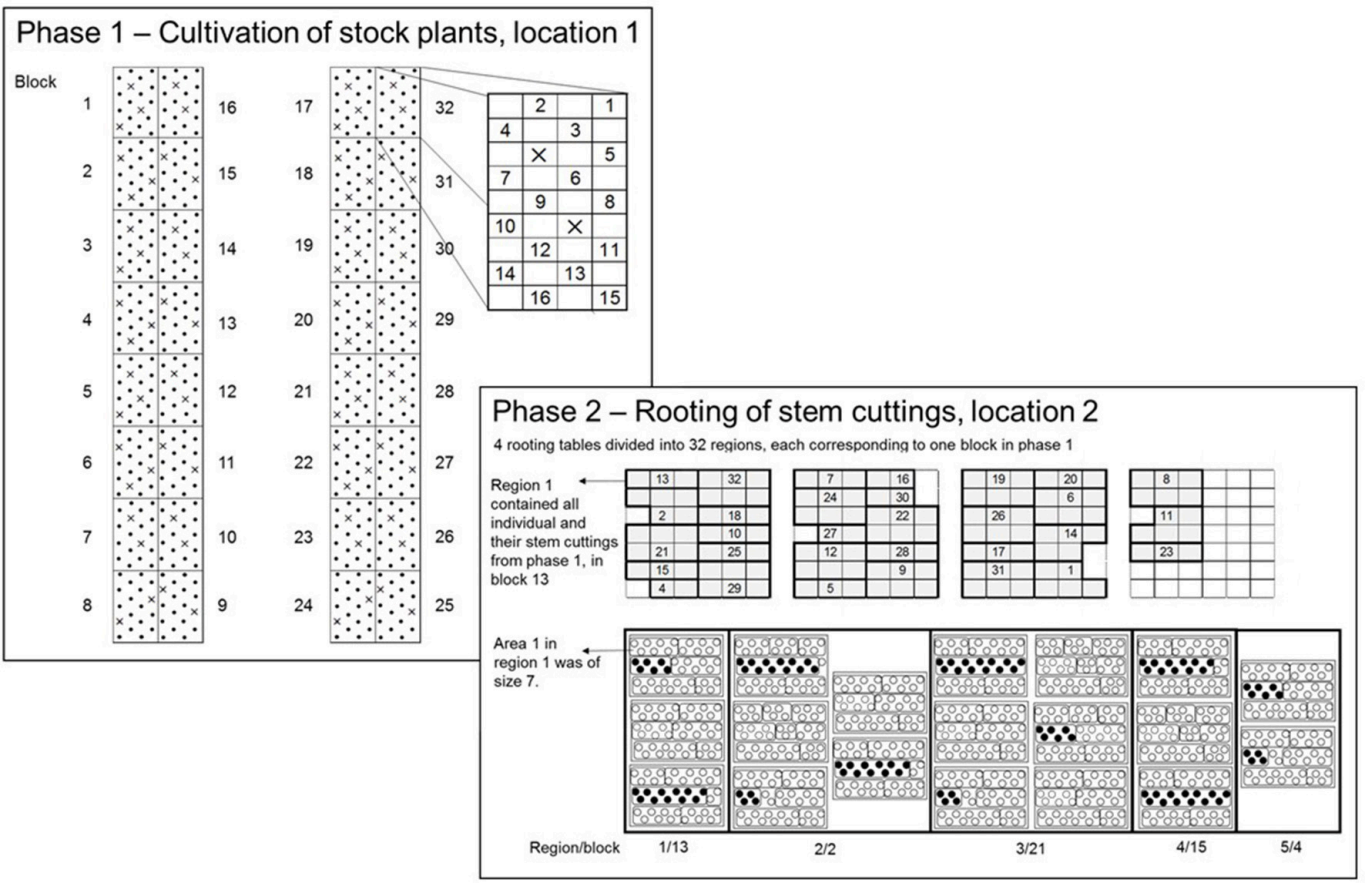
In P1, an augmented-design for 500 individuals in 32 blocks with 8 checks was used. Each dot represents an experimental unit in P1 (EU1) to which unreplicated entries were randomly allocated. On each EU1 a single stock plant of an individual from the seedling generation was placed. The crosses within blocks 1 to 32 indicate the check plots. In P2, the total experimental space was represented by four rooting tables. The four rooting tables were divided into 32 regions corresponding to blocks in P1 to conduct the randomization on-site. The numbers within regions correspond to block numbers in P1. Each region contained a variable number of trays depending on the number of SCC per individual within a block in P1. One tray contained 39 paper pots arranged in three rows, each with 13 paper pots. The trays were divided into areas, the experimental unit in P2 (EU2). The size of areas varied depending on the numbers of stem cuttings of and individual on each EU1. The planting of stem cuttings followed in a row-wise order.

In 2015, the two-phase experimental design was modified to assess individuals without replication, where the phases were as follows: Phase 1 (P1), the cultivation of stock plants of the seedling generation to obtain the SCC and Phase 2 (P2), the RF of stem cuttings (Figure 2). Each phase took place in a different greenhouse, but at the same location. The experimental design within each phase was an augmented design (Federer, 1956), in which the parental strains were tested with replications in incomplete blocks. The unreplicated individuals were randomly allocated to incomplete blocks. As will be described in more detail below, in P1, the experimental layout was generated by the SAS procedure OPTEX (SAS Institute Inc., 2014), whereas in P2 the randomization was carried out in the greenhouse on-site, because of biological matters of plant material.

##### Phase 1—augmented design −500 genotypes, 32 blocks, 8 checks

Using the OPTEX procedure (Piepho, 2015), an augmented design was generated for *c* = 8 checks, *v* = 500 genotypes in *b* = 32 blocks each of size 18 and a total 576 plots overall (Figure 2). Eight out of the ten parental genotypes were used as checks, except the parental strains “I” and “J.” The blocks were laid out on two cultivation tables, each comprising 16 blocks, leaving 38 free plots (experimental units; EU1) for checks in addition to the 250 plots for unreplicated entries. Since 38 is not a multiple of eight, there was space to replicate checks either nine or ten times. Because of lack of cuttings, checks could not be included in the experiment in the first phase, meaning that the plots intended for checks were empty in P1.

##### Phase 2—augmented design generation −500 genotypes, 32 blocks, 8 checks

To conduct the randomization on-site, four rooting tables were divided into 32 regions each corresponding to a single block in P1 (Figure 2; the numbers within regions in P2 correspond to block numbers in P1). Each region contained a variable number of trays depending on the obtained SCC of an individual in P1. One tray contained 39 paper pots arranged in three rows, each with 13 paper pots. The trays were divided into areas, the experimental unit in P2 (EU2). The randomization on-site was as follows: First, the checks were randomly allocated to regions and to areas within regions. Within the second step, the individuals were randomly allocated to the remaining areas within regions. Blocks of P1 were packaged as a single unit for transferal from P1 to P2. Note that all trays of a region fit on the same rooting table. The areas were filled in row-wise order on a tray and one area was planted directly following the previous, subject to the restriction that all paper pots for an area were on the same tray. The size of areas (EU2) varied depending on the SCC of an individual on an EU1.

#### Production-related traits

The SCC was assessed as the number of stem cuttings per single stock plant and genotype (either check or test individual) (EU1) in P1. The RF of stem cuttings of a single stock plant and genotype (either checks or test individual) was scored after four weeks of rooting. The number of plants in categories S0 (dead) to S5 (extraordinary) for each area (EU2) was counted (Molenaar et al., 2017). From these counts we computed the sum of rooted cuttings assigned to classes S4 and S5 so that a single response value was obtained per area (EU2).

#### Determining the best selection method by *H^2^*

*H*^2^ as described above was used to evaluate selection method for SCC and RF. The different selection methods were reflected by different LMM. The model for individual selection for SCC in the first phase, in symbolic form (Piepho et al., 2003; Piepho and Eckl, 2014), was

(1) $$\text{HR }:\text{ FM }\cdot \text{ ENTRY }+\text{ HR }\cdot \text{ WD }+\text{ HR }\cdot \text{ BLK }+\;\underline{\text{HR }\cdot \text{ BLK }\cdot \text{ PLT}},$$

where HR denotes the harvests, FM·ENTRY the individuals nested within families, HR·BLK the incomplete blocks nested within harvests, HR·WD the *post-blocking* factor “*worker-day*” nested within harvests, and HR·BLK·PLT, the residual error and experimental unit (plot = PLT) in P1 (EU1). The factor “*worker-day*” was defined to capture variation induced by working assistance of different people during the harvest of stem cuttings (Molenaar et al., 2018). Because of the unreplicated design, the estimation of the individuals within families-by-harvest interaction effect could not be achieved.

The model for family-index selection for SCC in P1, was an extension of model (1):

(2) $$\begin{array}{l} \text{HR }:\text{ FM }+\text{ FM }\cdot \text{ ENTRY }+\text{ HR }\cdot \text{ FM }+\text{ HR }\cdot \text{ WD}\\ \;+\text{ HR }\cdot \text{ BLK }+\;\underline{\text{HR }\cdot \text{ BLK }\cdot \text{ PLT}}, \end{array}$$

where FM denotes the families and HR·FM the family-by-harvest interaction. As explained above, family-index selection was implemented by fitting a CS variance-covariance structure for the sum of FM and FM·ENTRY random effects.

To evaluate the selection methods for RF in the second phase, some amendments to model (1) and (2) were necessary to account for checks, which were included in P2. Assuming individual selection, model (1) was changed to

(3) $$\begin{array}{l} \text{HR }+\text{ CK }+\text{ HR }\cdot \text{ CK }:\text{ PT }\cdot \text{ FM }\cdot \text{ ENTRY }+\text{ PT }\cdot \text{ HR }\cdot \text{ FM }\cdot \text{ ENTRY }\\ \;+\text{ HR }\cdot \text{ WD}+\text{ HR }\cdot \text{ BLK }+\;\underline{\text{HR }\cdot \text{ BLK }\cdot \text{PLT}} \end{array}$$

where CK is a factor for checks, comprising nine levels, i.e., eight levels for the parental strains (checks) and one level for the expected value of all individuals to separate effects of checks from individuals (Piepho et al., 2006). Furthermore, to prevent random genetic effects from being fitted for checks, a dummy variable PT with PT = 0 for checks and PT = 1 for individuals was defined. The dummy variable PT was crossed with the family and individuals within family effect. Similarly, model (2) was expanded by the check factor CK and the PT dummy variable to account for family-index selection for RF in P2,

(4) $$\begin{array}{l} \text{HR }+\text{ CK }+\text{ HR }\cdot \text{ CK }:\text{ PT }\cdot \text{ FM }+\text{ PT }\cdot \text{ FM }\cdot \text{ ENTRY }+\text{ PT }\cdot \text{ HR }\cdot \text{ FM}\\ \;+\text{ PT }\cdot \text{ HR }\cdot \text{ FM }\cdot \text{ ENTRY }+\text{ HR }\cdot \text{ WD }+\text{ HR }\cdot \text{ BLK }\\ \;+\;\underline{\text{HR }\cdot \text{ BLK }\cdot \text{ PLT}}. \end{array}$$

Family-index selection was implemented by fitting the CS variance-covariance structure to the sum of the PT · FM and PT · FM · ENTRY random effects.

### Phenotypic selection in ornamentals: the example of vase life assessment and related traits in *D. caryophyllus* L. breeding

This second example will illustrate the BLUP method for shelf-life and related traits in *D. caryophyllus* L., including vase life (VL) of cut flowers. The VL is one of the traits which most affects consumer satisfaction leading to repeated purchasing, and hence VL determines the economic value of a cultivar (Onozaki et al., 2001). Further, BLUP will be applied to floral traits such as flower size (FS) or number of buds (BN) and a morphology traits such as the stem length (SL). In 2016/2017 the entire seedling generation was cloned, so that each individual was tested by four replicates.

#### Plant material

Five crosses were made between ten elite *D. caryophyllus* L. strains belonging either to the mini or the standard carnation type to obtain five families segregating in the F_1_ already (Table 2). Families were assumed unrelated by pedigree. In total 176 individuals belonged to the standard type, and 328 individuals to the mini type, where three of the mini individuals were missing completely at random. The family sizes varied between 70 and 112 individuals.

**Table 2:** Parentage and size of the three mini carnation type and two standard carnation type families in D. *caryophyllus* L. evaluated in this study.

**Family**	***k* individuals in each family**	**Parental genotypes**			**Type**
		**paternal**		**maternal**	
1	106	(A	×	B)	*Mn*
2	110	(C	×	D)	*Mn*
3	112	(E	×	F)	*Mn*
1	106	(G	×	H)	*St*
2	70	(I	×	J)	*St*
*K_*total*_*	504				

#### Two-phase experimental design in *D. caryophyllus* breeding

For the assessment of vase life and related traits, the seedlings were clonally propagated 1 year in advance so that each individual was assessed by the use of four replications in the two-phase experimental set-up (Figure 2). In P1, the experimental layout was generated using CycDesigN 5.1 (VSN International, United Kingdom) and the experiment was conducted in the greenhouse, whereas in P2 the experiment was conducted in the lab, where the randomization was carried out on-site.

##### Phase 1 – resolvable incomplete block design with four replicates each 61 incomplete blocks of size 9 for 504 individuals and 45 check genotypes

**Figure 3:**
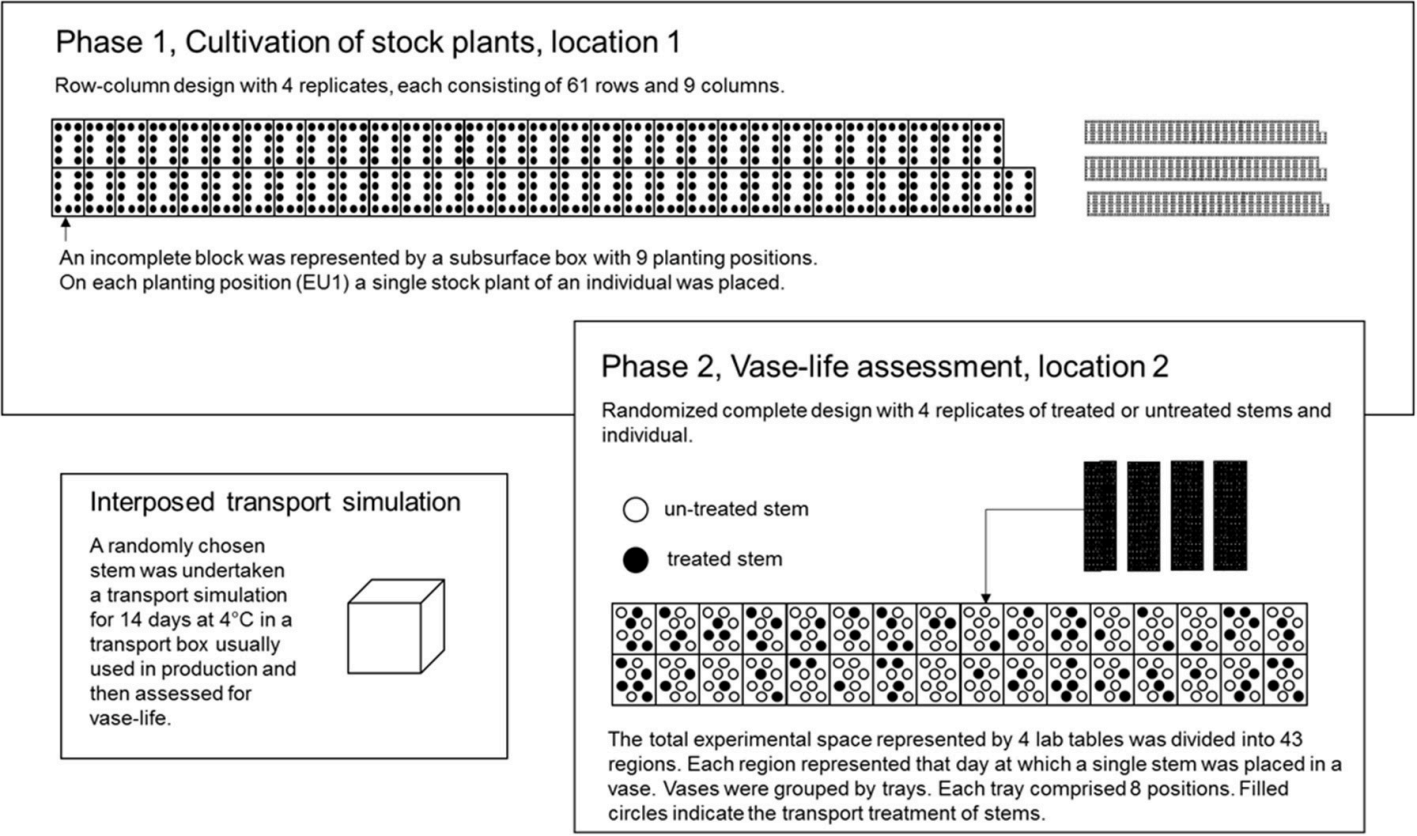
In P1, an incomplete block design for 504 individuals and 45 check genotypes in 61 blocks of size nine was used. The incomplete blocks were represented by subsurface boxes. Each dot in an incomplete block represents a planting position and hence, an experimental unit in P1 (EU1). On each EU1 a single stock plant of an individual from the seedling generation was placed. In P2, the total experimental space was represented by four cultivation tables. The four cultivation tables were divided into up 43 regions. Each region corresponds to that day on which a single stem of an EU1 was placed in a vase, independently, of whether the stem was storage treated or not. Furthermore, each day comprised several trays, each consisting of eight vases. A single vase represented the EU2. On each EU2 a single stem from EU1 was placed.

For each carnation type, a resolvable incomplete block design was generated by the use of CycDesigN 5.1 (VSN International, United Kingdom) with four replicates, each consisting 61 incomplete blocks of size nine. The incomplete blocks were represented by the physical units of subsurface boxes each consisting nine positions. The incomplete blocks of a replicate for both carnation types were jointly randomized, thus permitting a joint analysis of both types. Thus, a set of either nine standard or mini carnations was randomly allocated to each incomplete block. The randomization of genotypes was restricted in this way due to differences in cultivation minis with respect to flower bud removal. On each position of a subsurface box, i.e., on each experimental unit in P1 (EU1), a single stock plant of an individual was placed. Since 61 is not a multiple of 504, free positions were filled with up with another 45 check genotypes from another breeding program (Figure 3).

##### Phase 2 – randomized complete design for 504 individuals tested each with four replicates either treated or untreated

Because of biological matter (unpredictable development and maturity of flower buds of stock plants and individuals), a pre-defined design in the second phase was less suitable. That is why the total experimental space was divided up into 43 regions. Each region represented that day, on which a single stem of an individual and a replicate was placed in a vase. The vases were held by trays. Each tray comprised eight vases. A single vase represented the experimental unit in P2 (EU2). By the use of computer generated random numbers, first, the single stem of an individual and a replicate was randomly allocated to a tray within a region, and second, the stem was randomly allocated to a vase within a tray and region (EU2). The randomization of a stem was restricted, when the EU2 had been already filled with another individual's stem, in which case the stem was placed on the next empty EU2.

##### Interposed transport simulation between the two phases

Two stems were harvested from a single stock plant and individual (EU1). A randomly chosen stem was assessed immediately after harvesting in the laboratory for VL, whereas the other stem was first submitted to transport simulation for 14 days (Boxriker et al., 2017b) and afterwards assessed for VL in the lab.

#### Vase life and related traits

The total number of buds (BN) was counted on a single stem from a single stock plant and individual (EU1) for the mini carnation type. The stem length (SL) was assessed as the total length in centimeters (cm) of a single stem harvested from a single stock plant and EU1. The flower size (FS) was assessed as the diameter measured in cm of a single stem harvested from a single stock plant and individual in a vase (EU2) for the standard carnation type. The FS was measured of flowers that reached the fourth floral development stage (Figure 1, p. 63 in Boxriker et al., 2017a). The VL was assessed as the flower duration in the vase (EU2) in days of two stems from a stock plant and individual of EU1. For this purpose, stems were harvested at the second floral development stage (Figure 1, p. 63 in Boxriker et al., 2017a). One stem was randomly chosen and assessed immediately after harvesting in the laboratory for VL, whereas the other stem was submitted first to transport simulation for 14 days and then assessed for VL.

#### Determining the best selection method by *H^2^*

On the basis of the full two-phase model, reduced models were defined to simulate *H*^2^ for the traits BN and SL in P1, FS and VL in P2. Separate *H*^2^ for traits SL and VL for the mini and standard carnation were simulated, because the two carnation types belong to different subspecies of *D. caryphyllus* L. with different characteristics. The *H*^2^ assuming individual selection for SL either for mini or standard carnations was evaluated by the following model, listing fixed effects before the colon,

(5) $$\begin{array}{l} \text{REP}+\text{CK}+\text{STEM}+\text{TEMP}:\text{PT}\cdot \text{FM}\cdot \text{ENTRY}+\text{REP}\cdot \text{BLK}\\ \;+\underline{\text{REP}\cdot \text{BLK}\cdot \text{PLT}}, \end{array}$$

where REP denotes the replicates, CK a factor separating check genotypes, that were used to fill up empty positions in incomplete blocks and belonged either to mini or standard carnations from entries. Specifically, the CK factor comprised of 46 levels; 45 levels for the fillers and one single fixed effect to model the expected value of all individuals to separate effects of fillers from individuals. Furthermore, to prevent random effects from being fitted for fillers, a dummy variable PT with PT = 0 for fillers and PT = 1 for individuals was defined. TEMP was a covariate to account for the greenhouse temperature in P1, PT·FM·ENTRY denotes the individuals grouped by family effect, REP·BLK the incomplete blocks within replicates and the residual error REP·BLK·PLT. Expanding, model (5) by the term PT·FM, denoting the family effect, and implementing the CS structure for the sum of PT·FM and PT·FM·ENTRY random effects, family-index selection for SL was based on the model

(6) $$\begin{array}{l} \text{REP}+\text{CK}+\text{STEM}+\text{TEMP}:\text{PT}\cdot \text{FM}+\text{PT}\cdot \text{FM}\cdot \text{ENTRY}\\ \;+\text{REP}\cdot \text{BLK}+\underline{\text{ REP}\cdot \text{BLK}\cdot \text{PLT}}. \end{array}$$

Individual selection for BN for the mini carnation, was considered by expanding model (5) with a *post-blocking* factor POS to account better for variation induced by drop inlets of the sub-surface boxes (Boxriker et al., 2017b),

(7) $$\begin{array}{l} \text{REP}+\text{CK}+\text{STEM}+\text{TEMP}:\text{PT}\cdot \text{FM}\cdot \text{ENTRY}+\text{REP}\cdot \text{BLK}\\ \;+\text{REP}\cdot \text{POS}+\underline{\text{REP}\cdot \text{BLK}\cdot \text{PLT}}. \end{array}$$

The factor POS had nine levels, each represented one planting position within a subsurface box (Figure 2).

The family-index selection, model (6) was expanded by the *post-blocking* factor POS,

(8) $$\begin{array}{l} \text{REP}+\text{CK}+\text{STEM}+\text{TEMP}:\text{PT}\cdot \text{FM}+\text{PT}\cdot \text{FM}\cdot \text{ENTRY}\\ \;+\text{REP}\cdot \text{BLK}+\text{REP}\cdot \text{POS}+\underline{\text{REP}\cdot \text{BLK}\cdot \text{PLT}}. \end{array}$$

For the analysis of BN, the logarithm of the count data was used.

Individual selection in FS of standard carnation was implemented by extending model (5) with the terms DAY, DAY·VSE and STO,

(9) $$\begin{array}{l} \text{REP}+\text{STO}+\text{CK}+\text{STO}\cdot \text{CK}+\text{TEMP}:\text{PT}\cdot \text{FM}\cdot \text{ENTRY}\\ \;+\text{PT}\cdot \text{STO}\cdot \text{FM}\cdot \text{ENTRY}+\text{REP}\cdot \text{BLK}+\text{DAY}+\text{DAY}\cdot \text{VSE}\\ \;+\underline{\text{REP}\cdot \text{BLK}\cdot \text{PLT}}, \end{array}$$

where STO denote the transport simulation (yes/no), STO·CK the check genotype-by-transport interaction, PT·STO·FM·ENTRY the individual-by-transport interaction, DAY the block when a single stem of an individual and position was and DAY·VSE the positional effect of a vase within a day. Family-index selection was implemented by adding the family effect and the family-by-transport interaction to model (9),

(10) $$\begin{array}{l} \text{REP}+\text{STO}+\text{CK}+\text{STO}\cdot \text{CK}+\text{TEMP}:\text{PT}\cdot \text{FM}\\ \;+\text{PT}\cdot \text{FM}\cdot \text{ENTRY}+\text{PT}\cdot \text{STO}\cdot \text{FM}+\text{PT}\cdot \text{STO}\cdot \text{FM}\cdot \text{ENTRY}\\ \;+\text{REP}\cdot \text{BLK}+\text{DAY}+\text{DAY}\cdot \text{VSE}+\underline{\text{REP}\cdot \text{BLK}\cdot \text{PLT}}. \end{array}$$

For the sum of random effects PT· FM and PT· FM· ENTRY of model (10) the CS structure was fitted.

Individual selection in VL for mini or standard carnation was performed by,

(11) $$\begin{array}{l} \text{REP}+\text{STO}+\text{CK}+\text{STO}\cdot \text{CK}+\text{TEMP}:\text{PT}\cdot \text{FM}\cdot \text{ENTRY}\\ \;+\text{PT}\cdot \text{STO}\cdot \text{FM}\cdot \text{ENTRY}+\text{REP}\cdot \text{BLK}+\text{REP}\cdot \text{POS}\\ \;+\text{DAY}+\underline{\text{REP}\cdot \text{BLK}\cdot \text{PLT}}, \end{array}$$

whereas family-index selection was implemented for by

(12) $$\begin{array}{l} \text{REP}+\text{STO}+\text{CK}+\text{STO}\cdot \text{CK}+\text{TEMP}:\text{PT}\cdot \text{FM}\\ \;+\text{PT}\cdot \text{FM}\cdot \text{ENTRY}+\text{PT}\cdot \text{STO}\cdot \text{FM}\\ \;+\text{PT}\cdot \text{STO}\cdot \text{FM}\cdot \text{ENTRY}+\text{REP}\cdot \text{BLK}+\text{REP}\cdot \text{POS}\\ \;+\text{DAY}+\underline{\text{REP}\cdot \text{BLK}\cdot \text{PLT}}, \end{array}$$

and the CS structure was fitted for the sum of random effects PT·FM and PT·FM·ENTRY.

## Results

In *P. zonale*, the highest *H*^2^ was always found for family-index selection. In *D. caryophyllus* L. the *H*^2^ was approximately the same for individual and family-index selection for BN, SL and VL in standard carnations. *H*^2^ was greater for family-index selection for FS in standard carnation and VL in mini carnation (Table 3). The results were supported by the variance component estimates and box plots of BLUPs, which are described in detail below.

**Table 3:** Evaluation of selection methods in terms of simulated heritability and corresponding standard errors.

**Species**	**Trait**	**CT**^†^	**Selection methods**			
			**Individual selection**		**Family-index selection**	
			***r^**2**^***	***s*.*e*.(*r*^2^)**	***r^**2**^***	***s*.*e*.(*r*^2^)**
*P.zonale*	SCC		0.3376	0.003394	0.3718	0.003783
	RF		0.4112	0.004126	0.5601	0.005672
*D. caryophyllus* L.	BN	*Mn*	0.6904	0.006910	0.6970	0.006986
	SL	*Std*	0.9490	0.009491	0.9447	0.009449
		*Mn*	0.8911	0.008912	0.8913	0.008914
	FS	*Std*	0.6458	0.006473	0.6561	0.006583
	VL	*Std*	0.7118	0.007128	0.7128	0.007147
		*Mn*	0.7688	0.007692	0.7752	0.007817

† *Carnation type*.

### Variance component estimates from the *P. zonale* breeding

The genotypic variance component estimate (FM·ENTRY) for both SCC and RF was relatively low in proportion to the total variation (Tables 4, 5), which is also reflected by the shrinkage property of BLUPs toward the general mean (zero reference line in Presentation 2).

**Table 4:** Variance components of random effects obtained from model (1) and (2) to evaluate the individual and family-index selection for stem cutting count (SCC).

**Model term**	**Variance**	
	**Model (1)**	**Model (2)**
FM	–	0.2121
HR·FM	–	0.1481
FM·ENTRY	1.0835	0.8727
HR·BLK	2.3069	2.3397
HR·WD	0.7977	0.8456
HR·BLK·PLT	5.4417	5.3129

**Table 5:** Variance component estimates of random effects obtained from model (3) and (4) to evaluate individual and family-index selection for root formation (RF).

**Model term**	**Variance**	
	**Model (3)**	**Model (4)**
FM	–	1.0818
HR·FM	–	0.2108
FM·ENTRY	1.9071	1.7697
HR·FM·ENTRY	4.1932	3.9096
HR·BLK	0.9126	0.9508
HR·WD	2.0586	0.9006
HR· BLK· PLT	2.9356	2.9598

In P1, by far the largest variance component was the residual error variance, followed by the block variance for analyzing the SCC (Table 4). Similar variance components for the residual error variance and the block effects were calculated in a former experiment in 2013/14, although genotypes in that experiment were tested in four replications (Molenaar et al., 2017). Moreover, under the assumption of family-index selection, the effect for the family-by-harvest interaction (HR·FM) was found to be small, and the residual error variance was reduced. This suggested that the unaccounted for serial correlation on the same plots within blocks and harvest might have inflated the small genotypic and the residual error variance. The worker-induced (HR·WD) and the genotypic (FM·ENTRY) variances were of comparable size, indicating the considerable effect of the person carrying out the assessment of production-related traits (Molenaar et al., 2017).

In contrast to P1, in P2 the largest variance component was calculated for the individual within family-by-harvest interaction effect (HR·FM·ENTRY) and was greater than the residual error variance (Table 5). The interaction between genotypes and harvests had already been observed and discussed in 2013/14 (Molenaar et al., 2017). Reasons were attributed to environmental conditions such as change in day length during the experimentation or cultivation management, in particular the watering. Similar to P1, the variance of the average “*worker-day*” effect had almost the same size as the variance of the FM·ENTRY effect. The genotypic variance for RF was approximately twice as high as for SCC and also the family effect for RF was greater than for SCC.

In box plots, the relatively low genotypic variance for SCC and RF became visible by the shrinkage property of BLUP in cases when genotypic variation was low or missing; the BLUPs are then all shrunken toward the general mean (Appendix Presentation 2 in Supplementary Material). As BLUP assumes a normal distribution with zero mean, the zero reference line represents the zero on the y-axis in comparing box plots for the two selection methods. Assuming individual selection the BLUPs for SCC and RF were close to zero, except for Families 1 and 5. In contrast, by accounting for family-information, a ranking between families was notable. Furthermore, the increased accuracy of BLUPs when accounting for family information is illustrated also by the shortened whiskers of boxes in box plots for family-index selection in comparison to box plots for individual selection (Appendix Presentation 2 in Supplementary Material).

Generally, the selection based on BLUP for SCC and RF would be reasonable, because the Q-Q plots of standardized BLUPs for SCC and RF revealed that random genotypic effects were approximately normal as required (Appendix Presentation 3 in Supplementary Material).

### Variance component estimates from the *D. caryphyllus* L. breeding

The genotypic variance component estimate for FM·ENTRY for BN, SL, FS, and VL was almost always relatively high in proportion to the total variation (Tables 6–**9**) and hence, large simulated *H*^2^ were obtained for shelf-life traits in comparison to the simulated *H*^2^ for production-related traits *P. zonale* breeding.

**Table 6:** Variance component estimates of random effects obtained from model (5) and (6) to evaluate individual and family-index selection for stem length (SL).

**Model term**	**Variance component estimates**			
	**Mini carnation**		**Standard carnation**	
	**Model (5)**	**Model (6)**	**Model (5)**	**Model (6)**
FM	–	4.1724	–	69.3944
FM·ENTRY	56.8179	53.9425	111.18	78.0894
REP·BLK	1.6502	1.6067	1.8719	1.4208
REP·BLK·PLT	31.8940	31.9223	34.2640	34.3225

**Table 7:** Variance component estimates of random effects obtained from model (7) and (8) to evaluate individual and family-index selection for bud number (BN).

**Model term**	**Variance component estimates**^†^	
	**Model (7)**	**Model (8)**
FM	-	0.0128
FM·ENTRY	0.0581	0.0486
REP·BLK	0.0030	0.0029
REP·POS	0.0349	0.0353
REP·BLK·PLT	0.0695	0.0695

† *Log-transformed*.

The different characteristics between mini and standard carnation with respect to SL became apparent in particular when family-index selection was considered. The family variance component estimate for SL of mini carnation was negligibly small in comparison to the individual variance component estimate, indicating that families of mini carnation vary less for SL than individuals vary within families. In contrast, standard carnation families differ greatly for SL, as indicated by individual variance component estimates similar to family variance component estimate. Mini and standard carnation differ greatly in size, the minis being smaller than standards. For both carnation types the variance of incomplete block effects was marginal in comparison to the genotypic variance components (FM and FM·ENTRY) or the residual error variance.

Another P1 trait was BN for the mini carnation type. Also for this trait, the family variance component estimate was much smaller than the individual variance component estimate. By far the smallest variance component estimate was found for incomplete blocks. The variance component estimate of the *post*-*blocking* positional effect within incomplete blocks was much greater suggesting that variation in water supply influences the development of BN per single stem and stock plant per position.

In P2, for FS in standards by far the smallest genotypic variance component estimate for FM·ENTRY was obtained and accordingly the smallest *H*^2^ was simulated for this shelf-life trait (Table 8). Moreover, the FS of individuals was not affected by the interposed transport simulation, indicated by the zero variance component estimate for the random family-by-transport interaction effect (STO·FM) or individual-by-transport interaction effect (STO·FM·ENTRY). No or only a small proportion of the environmental variation was captured by the incomplete block effect in P1 or by the day block effect and the positional effect in P2, where the residual error variance was the largest variance component estimate and the family-effect for FS was smaller than that for the positional effect.

**Table 8:** Variance component estimates of random effects obtained from model (9) and (10) to evaluate individual and family-index selection method for flower size (FS).

**Model term**	**Variance component estimates**	
	**Model (9)**	**Model (10)**
FM	–	0.0249
FM·ENTRY	0.1409	0.1305
STO·FM	–	0
STO·FM·ENTRY	0	0
REP·BLK	0	0
DAY	0.0056	0.0055
DAY·VSE	0.0421	0.0730
REP·BLK·PLT	0.2347	0.2034

The different characteristic between mini and standard carnation became apparent again for the VL assessed for both carnation types. In particular both carnation types varied for individual and family effects and for individual-by-transport interaction effect. A much greater genotypic (FM·ENTRY) variance component for VL was found for mini carnation, which was almost as large as the residual error variance component estimate when individual selection was considered (Table 9). The genotypic (FM·ENTRY) variance component estimate was half the size for that of standards when individual selection was considered. When family information was exploited, the largest genotypic variation was observed for the family effect (FM) in mini carnation indicating that the families varied strongly for VL. However, the family-by-transport interaction effect was the smallest variance component estimate when considering family-index selection for mini carnations, beside the incomplete block effect variance which was estimated to be zero. The individual effect variance was half the size of the family effect variance, however, for minis a relatively large variance for the individual-by-transport interaction effect was estimated either under individual or family index selection. This interaction effect variance was estimated to be zero for the standard carnation type, although the estimated family-by-transport interaction effect variance was similar to that for the mini carnation type. The variance of the family effect for standard carnations was much smaller in comparison to that for the individual effect. Interestingly, also the environmental conditions seemed to affect the VL differently. The effect of incomplete blocks was much smaller for the mini carnations than for the standard carnations, where the variance of the *post-blocking* effect in P1 was of similar size. But the day effect for mini carnations was much greater than for the standard carnations.

**Table 9:** Variance component estimates of random effects obtained from model (11) and (12) to evaluate individual and family-index selection for vase life (VL).

**Model term**	**Variance component estimates**			
	**Mini carnation**		**Standard carnation**	
	**Model (11)**	**Model (12)**	**Model (11)**	**Model (12)**
FM	–	3.9604	–	0.7040
FM·ENTRY	4.6019	1.8754	2.4475	2.0988
STO·FM	–	0.0320	–	0.0247
STO·FM·ENTRY	0.4432	0.4439	0	0
REP·BLK	0.0275	0	0.1788	0.1736
REP·POS	0.6453	0.6829	0.6522	0.6419
DAY	0.3015	0.3443	0.0658	0.0705
REP·BLK·PLT	4.4935	4.4823	4.8872	4.8909

The selection based on BLUPs for shelf-life traits would be reasonable, because Q-Q plots of standardized BLUPs for shelf-life traits revealed no departure from normality (Appendix Presentation 2 in Supplementary Material). This is also evidenced by the box plots of BLUPs of the shelf-life traits, except for VL in mini carnation for family-index selection (Appendix Presentation 3 in Supplementary Material). The standardized BLUPs showed a bimodal distribution. However, the shrinkage property of BLUP was not as strong as for the production-related traits, because the individual effect almost always had the largest variance component estimate under individual selection. Hence, changes in ranks between individual and family-index selection was not as pronounced as for production-related traits.

## Discussion

Plant breeders always face the challenge to select the best individuals. Selection methods are required that maximize the use of available data (Bernardo, 2010) and greater selection gain can be expected when methods accounting for pedigree structure are employed (Piepho and Williams, 2006). The BLUP procedure achieves this by combining phenotypic data with information on pedigree relationships (Bernardo, 2010). A selection method that exploit family information is the family-index selection, which is at least as efficient as individual selection (Lynch and Walsh, 2013). This was confirmed by *D. caryophyllus* L., except for FS in standard carnation and VL in mini carnation.

### When family-index selection is the better choice

Family-index selection is the better choice for traits with low heritability, which was confirmed for *P. zonale* (Table 3). The simulated *H*^2^ for the family-index selection was always upwards from four units better than for the individual selection, which may be explained by the exploitation of relationships of relatives. When family-index selection outperformed individual selection, the total genetic variance was higher than under individual selection, whereas changes of variance component estimates of random block effects or the residual error variance were not as succinct (Tables 4, 5, 7, 9). In relation to Lush's example, the individual effects were shrunken toward the family means rather than the overall mean by the use of the intra-class correlation coefficient (Appendix Presentation 2 in Supplementary Material). As a result, the family means were estimated with higher accuracy and differences between families become more obvious. Best performing individuals of the superior Family 1 remained best. Best performing individuals of poor families were shrunken toward the lower family mean and remained no longer in the selected fraction. Thus, the main difference in individual and family-index selection lies in the adjustment of estimating the individual's effects depending on the estimated variance component of random individual and family effects in a breeding trial, i.e., on the intra-class correlation coefficient. By making this adjustment, the superior performance of family-index depends not only on the ratio between total genotypic and residual error variances, but also on the ratio between the family and individual variances of the total genetic variance. Pure family selection was not considered, because individual performances between families will almost always overlap (Appendix Presentation 2 in Supplementary Material).

### More on exploiting the information of relatives

It is well known that floral characteristics, growth characteristics and cultivation methods differ between mini and standard carnation types. However, such strong differences of the individual performances depending on the family background were unexpected for SL and VL (Table 3) implying different strategies in breeding. For example in mini carnations, the performance of individuals on SL is almost totally independent of the family background (*t* = 0.07), whereas for VL a high intra-class correlation coefficient was found (*t* = 0.68). The high intra-class correlation coefficient means that individuals within families are more similar than across families. This indicates the importance of selecting parental strains used for crosses for VL improvement, which can be further investigated by the general or specific combining ability for example. This is contrary to the dependence of individual performance for SL and VL on the family background in standard carnations, which reveals that in *D. caryophyllus* L. the two carnation types should be bred in different programs. Thus, exploiting the information of relatives the genetics underlying the traits must be observed.

### BLUP in ornamentals

In clonal breeding, the greatest genetic variability exists in the seedling generation (Figure 1 in Molenaar et al., 2017). Each seedling and individual is represented by a single plant. Experimental designs that are suitable to test unreplicated treatments are augmented designs, as applied in *P. zonale*. Block effects are estimated solely by the use of checks, which might not capture all environmental variation on the estimation of the treatments effects. In the seedling generation, the only way to increase the precision of estimating treatment effects is to exploit the information of relatives, confirmed by *P. zonale* (Tables 3, 4). In the primary selection of seedlings, the population size is the drastically reduction of from thousands to a maximum of 200 individuals. From that primary selection until the official testing, selected individuals are only clonally propagated.

In the clonal generations, individual genotypes can be tested in replications, for example in resolvable incomplete block designs, as applied in *D. caryophyllus* L. or in randomized complete block designs in later breeding stages as the number of individuals is more and more reduced (Figure 1 in Molenaar et al., 2017). Higher precision of estimated treatment effects can be expected, because the block effects are estimated from the individuals included in all replicates. However, as the population size is reduced, the genetic variability is reduced, too, from the seedling to the first clonal generation. Differences between individuals might become difficult to detect. But here too, consideration of family information may improve treatment estimates. In the present study, the effect of reduced genetic variability and the use of a replicated individuals could not be investigated, as the entire seedling generation was clonally propagated before the vase life tests, which is rarely done in practice.

Furthermore, if families in clonal generations are tested in different locations, each genetically identical individual within a family can be tested across locations, and hence, each individual is replicated across locations. This allows a precise determination of genotype-by-environment interaction. By comparison, with non-clonable species, only families can be replicated across locations, but not individuals within families.

## Conclusion

The choice of a selection method has implications for selection gain. Family-index selection was found to be at least as efficient as individual selection, surpassing the efficiency of individual selection when the heritability was low. Another important aspect for breeders is the shrinkage property of the family-index selection, where superior individuals are shrunken downwards and inferior individuals are shrunken upwards, yielding in a change of genotype ranks protecting to do false selection decision. Furthermore, exploiting the information of relatives can be used to investigate the genetics behind traits and reveal strategies for selecting parental strains for crosses. Our results support the need for separating the breeding program for *D. caryophyllus* L. into mini and standard types. The present work illustrated the use of BLUP in *P. zonale* and *D. caryophyllus* L., which are exemplary for ornamental and clonal breeding in general.

## Author contributions

HM conceived and participated in the design of the study, conducted the analysis, interpreted the results and prepared the manuscript. H-PP participated in the design of the study, in its writing and editing of the manuscript and oversaw the project. H-PP and RB revised the manuscript. All authors discussed the results, commented on the manuscript and have approved the final manuscript.

### Conflict of interest statement

The authors declare that the research was conducted in the absence of any commercial or financial relationships that could be construed as a potential conflict of interest.

## Abbreviations

BLUP: best linear unbiased prediction
BLUE: best linear unbiased estimation
LMM: linear mixed model
SCC: stem cutting count
CS: compound symmetry
EU1: the experimental unit in P1
EU2: the experimental unit in P2
P1: phase one
P2: phase two
RF: root formation
VL: vase life
FS: flower size
BN: bud number
SL: stem length.

## References

[B1] Barbosa M. Resende M. Bressiani J. Silveira L. Peternelli L. (2005). Selection of sugarcane families and parents by Reml/Blup. Crop Breed. Appl. Biotechnol. 5, 443–450. 10.12702/1984-7033.v05n04a10

[B2] Bernardo R. (2010). Breeding for Quantitative Traits in Plants. Woodbury, MN: Stemna Press.

[B3] Borges V. Ferreira P. V. Soares L. Santos G. M. Santos A. M. M. (2010). Seleção de clones de batata-doce pelo procedimento REML/BLUP. Acta Scientiarum Agronomy 32, 643–649. 10.4025/actasciagron.v32i4.4837

[B4] Boxriker M. Boehm R. Krezdorn N. Rotter B. Piepho H. P. (2017a). Comparative transcriptome analysis of vase life and carnation type in *Dianthus caryophyllus* L. Sci. Hortic. 217, 61–72. 10.1016/j.scienta.2017.01.015

[B5] Boxriker M. Boehm R. Möhring J. Piepho H. P. (2017b). Efficient statistical design in two-phase experiments on vase life in carnations (*Dianthus caryophyllus* L.). Postharvest Biol. Technol. 128, 161–168. 10.1016/j.postharvbio.2016.12.003

[B6] Copas J. (1983). Regression, prediction and shrinkage. J. R. Statist. Soc. Series B. 45, 311–354.

[B7] Cullis B. R. Smith A. B. Coombes N. E. (2006). On the design of early generation variety trials with correlated data. J. Agric. Biol. Environ. Stat. 11, 381–393. 10.1198/108571106X154443

[B8] Falconer D. S. Mackay T. F. C. (1996). Introduction to Quantitative Genetics. London: Prentice Hall. 10.1093/genetics/167.4.1529 PMC1471025 15342495

[B9] Federer W. T. (1956). Augmented (or hoonuiako) designs. Hawaiian Planter Record 55, 191–208.

[B10] Fogaça L. A. Oliveira R. A. Cuquel F. L. Filho J. C. B. Vendrame W. A. Tombolato A. F. C. (2012). Heritability and genetic correlation in daylily selection. Euphytica 184, 301–310. 10.1007/s10681-011-0478-y

[B11] Gianola D. Cecchinato A. Naya H. Schön C.-C. (2018). Prediction of complex traits: robust alternatives to best linear unbiased prediction. Front. Genet. 9:195. 10.3389/fgene.2018.00195 29951082 PMC6008589

[B12] Graser H. Smith S. Tier B. (1987). A derivative-free approach for estimating variance components in animal models by restricted maximum likelihood. J. Anim. Sci. 64, 1362–1370. 10.2527/jas1987.6451362x

[B13] Henderson C. R. (1950). Estimation of genetic parameters. Ann. Math. Statist. 21, 309–310.

[B14] Hill R. Rosenberger J. (1985). Methods for combining data from germplasm evaluation trials. Crop Sci. 25, 467–470. 10.2135/cropsci1985.0011183X002500030009x

[B15] Holland J. B. Nyquist W. E. Cervantes-Martínez C. T. (2003). Estimating and interpreting heritability for plant breeding: an update. Plant Breed. Rev. 22, 9–112. 10.1002/9780470650202.ch2

[B16] Huang H. Harding J. Byrne T. Famula T. (1995). Estimation of long-term genetic improvement for gerbera using the best linear unbiased prediction (BLUP) procedure. Theor. Appl. Genet. 91, 790–794. 10.1007/BF00220961 24169918

[B17] Legarra A. (2016). Comparing estimates of genetic variance across different relationship models. Theor. Popul. Biol. 107, 26–30. 10.1016/j.tpb.2015.08.005 26341159

[B18] Lehermeier C. de los Campos G. Wimmer V. Schön C.-C. (2017). Genomic variance estimates: with or without disequilibrium covariances? J. Anim. Breed. Genet. 134, 232–241. 10.1111/jbg.12268 28508483

[B19] Lush J. L. (1947). Family merit and individual merit as bases for selection. Part I Am. Nat. 81, 241–261. 10.1086/281520

[B20] Lynch M. Walsh B. (2013). Genetics and Analysis of Quantitative Traits. Sunderland, M. A: Sinauer Associates, Inc.

[B21] Misztal I. Gianola D. (1987). Indirect solution of mixed-model equations. J. Diary Sci. 70, 716–723. 10.3168/jds.S0022-0302(87)80063-2

[B22] Molenaar H. Boehm R. Piepho H. P. (2018). Identifying effective design approaches to allocate genotypes in two-phase designs: a case study in *Pelargonium zonale*. Front. Plant Sci. 8:2194. 10.3389/fpls.2017.02194 29354145 PMC5760546

[B23] Molenaar H. Glawe M. Boehm R. Piepho H. P. (2017). Selection for production-related traits in Pelargonium zonale: improved design and analysis make all the difference. Horticult. Res. 4:17004. 10.1038/hortres.2017.4 28243453 PMC5321157

[B24] Oakey H. Verbyla A. P. Cullis B. R. Wei X. Pitchford W. S. (2006). Joint modeling of additive and non-additive (genetic line) effects in multi-environment trials. Theoret. Appl. Genet. 114, 1319–1332. 10.1007/s00122-007-0515-3 17426958

[B25] Onozaki T. Ikeda H. Yamaguchi T. (2001). Genetic improvement of vase life of carnation owers by crossing and selection. Sci. Hortic. 87, 107–120. 10.1016/S0304-4238(00)00167-9

[B26] Piepho H. P. (1994). Best Linear Unbiased Prediction (BLUP) for regional yield trials: a comparison to additive main effects and multiplicative interaction (AMMI) analysis. Theoret. Appl. Genet. 89, 647–654. 10.1007/BF00222462 24177943

[B27] Piepho H. P. (2015). Generating efficient designs for comparative experiments using the SAS procedure OPTEX. Commun. Biomet. Crop Sci. 10, 96–114.

[B28] Piepho H. P. Büchse A. Emrich K. (2003). A hitchhiker's guide to mixed models for randomized experiments. J. Agronomy Crop Sci. 189, 310–322. 10.1046/j.1439-037X.2003.00049.x

[B29] Piepho H. P. Eckl T. (2014). Analysis of series of variety trials with perennial crops. Grass Forage Sci. 69, 431–440. 10.1111/gfs.12054

[B30] Piepho H. P. Möhring J. (2007). Computing heritability and selection response from unbalanced plant breeding trials. Genetics 177, 1881–1888. 10.1534/genetics.107.074229 18039886 PMC2147938

[B31] Piepho H. P. Möhring J. Melchinger A. E. Büchse A. (2008). BLUP for phenotypic selection in plant breeding and variety testing. Euphytica 161, 209–228. 10.1007/s10681-007-9449-8

[B32] Piepho H. P. Williams E. R. (2006). A comparison of experimental designs for selection in breeding trials with nested treatment structure. Theoret. Appl. Genet. 113, 1505–1513. 10.1007/s00122-006-0398-8 17028902

[B33] Piepho H. P. Williams E. R. Fleck M. (2006). A note on the analysis of designed experiments with complex treatment structure. HortScience 41, 446–452.

[B34] Resende M. (2016). Software Selegen-REML/BLUP: a useful tool for plant breeding. Crop Breed. Appl. Biotechnol. 16, 330–339. 10.1590/1984-70332016v16n4a49

[B35] Robinson G. K. (1991). That BLUP is a good thing: the estimation of random effects. Statist. Sci. 6,15–51. 10.1214/ss/1177011926

[B36] Santos E. A. Viana A. P. de Oliveira Freitas J. C. Rodrigues D. L. Tavares R. F. Paiva C. L. (2015). Genotype selection by REML/BLUP methodology in a segregating population from an interspecific *Passiflora* spp. crossing. Euphytica 204, 1–11. 10.1007/s10681-015-1367-6

[B37] Searle S. R. Casella G. McCulloch C. E. (1992). Variance Components. Hoboken, NJ: John Wiley &Sons. 10.1002/9780470316856

[B38] Singh B. D. Singh A. K. (2015). Marker-Assisted Plant Breeding: Principles and Practices. Heidelberg; New York, NY, Dordrecht, London: Springer 10.1007/978-81-322-2316-0

[B39] Slater A. T. Wilson G. M. Cogan N. O. I. Forster J. W. Hayes B. J. (2014). Improving the analysis of low heritability complex traits for enhanced genetic gain in potato. Theoret. Appl. Genet. 127, 809–820. 10.1007/s00122-013-2258-7 24374468

[B40] Teixeira D. H. L. Oliveira M. S. P. Goncalves F. M. A. Nunes J. A. R. (2012). Selection index for simultaneously improving fruit production components of assai palm. Pesq. Agropec. Brasil. 47, 237–243. 10.1590/S0100-204X2012000200012

[B41] Ticona-Benavente C. A. da Silva Filho D. F. (2015). Comparison of BLUE and BLUP/REML in the selection of clones and families of potato (Solanum tuberosum). Genet. Mol. Res. 14, 18421–18430. 10.4238/2015.December.23.30 26782490

[B42] Van Raden P. M. (2008). Efficient methods to compute genomic predictions. J. Diary Sci. 91, 4414–4423. 10.3168/jds.2007-0980 18946147

[B43] Wang B. Sverdlov S. Thompson E. (2017). Efficient estimation of realized kinship from single nucleotide polymorphism genotypes. Genetics 205, 1063–1078. 10.1534/genetics.116.197004 28100587 PMC5340323

[B44] Zeni Neto H. Daros E. Bespalhok Filho J. C. Scapim C. A. Vidigal M. C. G. Vidigal Filho P. S. (2013). Selection of families and parents of sugarcane (Saccharum spp.) through mixed models by joint analysis of two harvests. Euphytica 193, 391–408. 10.1007/s10681-013-0947-6

