# Supplementary material for: Phenotypic Selection in Ornamental Breeding: It's Better to Have the BLUPs Than to Have the BLUEs
Source: Front Plant Sci. 2018 Nov 5;9:1511. doi: 10.3389/fpls.2018.01511 (PMC6230591; doi:10.3389/fpls.2018.01511)
Supplement: Supplementary file 1 [file Data_Sheet_1.PDF]

Unequal family sizes

We assumed that in each family the same number of individuals were assessed for SCC in the motivating example, which is an idealized condition rather than a reality. In a real breeding program, the amount of seed per performed cross may vary, seedling emergence and survival rate depending on various environmental conditions. Thus, the number of individuals varies among families when raising plant material for phenotyping. The unequal family sizes matter for the prediction of BLUPs by the use of certain statistical programs, therefore the $\boldsymbol{G}$ matrix is needed to be decomposed according to,

$$\boldsymbol{G} = \boldsymbol{V}_1 \cdot \sigma_f^2 + \boldsymbol{V}_2 \cdot \sigma_s^2,$$

where $\boldsymbol{V}_1$ and $\boldsymbol{V}_2$ are coefficient matrices for the families and individuals within families. On the $\boldsymbol{V}_1$ diagonal are $\boldsymbol{J}_{n_i}$ all-ones matrices and on the $\boldsymbol{V}_2$ diagonal are $\boldsymbol{I}_{n_i}$ identity matrices, where the indices n_i denotes the family size n of the i-th family and hence the dimension of the square matrices $\boldsymbol{J}$ and $\boldsymbol{I}$

$$\boldsymbol{G} = \begin{pmatrix} \boldsymbol{J}_{n_i} & 0 & 0 & 0 \\ 0 & \boldsymbol{J}_{n_i} & 0 & 0 \\ 0 & 0 & \boldsymbol{J}_{n_i} & 0 \\ 0 & 0 & 0 & \boldsymbol{J}_{n_i} \end{pmatrix} \cdot \sigma_f^2 + \begin{pmatrix} \boldsymbol{I}_{n_i} & 0 & 0 & 0 \\ 0 & \boldsymbol{I}_{n_i} & 0 & 0 \\ 0 & 0 & \boldsymbol{I}_{n_i} & 0 \\ 0 & 0 & 0 & \boldsymbol{I}_{n_i} \end{pmatrix} \cdot \sigma_s^2.$$

The i = 1 to 4 family specific variance-covariance matrix is obtained in a linear manner,

$$\boldsymbol{G}_\mathrm{i} = \boldsymbol{J}_{n_i} + \boldsymbol{I}_{n_i},$$

$$\boldsymbol{G}_\mathrm{i} = \begin{pmatrix} 1 & 1 & 1 & 1 \\ 1 & 1 & 1 & 1 \\ 1 & 1 & 1 & 1 \\ 1 & 1 & 1 & 1 \end{pmatrix} \sigma_f^2 + \begin{pmatrix} 1 & 0 & 0 & 0 \\ 0 & 1 & 0 & 0 \\ 0 & 0 & 1 & 0 \\ 0 & 0 & 0 & 1 \end{pmatrix} \sigma_s^2.$$
