# Supplementary material for: Phenotypic Selection in Ornamental Breeding: It's Better to Have the BLUPs Than to Have the BLUEs
Source: Front Plant Sci. 2018 Nov 5;9:1511. doi: 10.3389/fpls.2018.01511 (PMC6230591; doi:10.3389/fpls.2018.01511)
Supplement: Supplementary file 2 [file Data_Sheet_2.PDF]

Dianthus caryophyllus L.

Standard carnations

Flower size

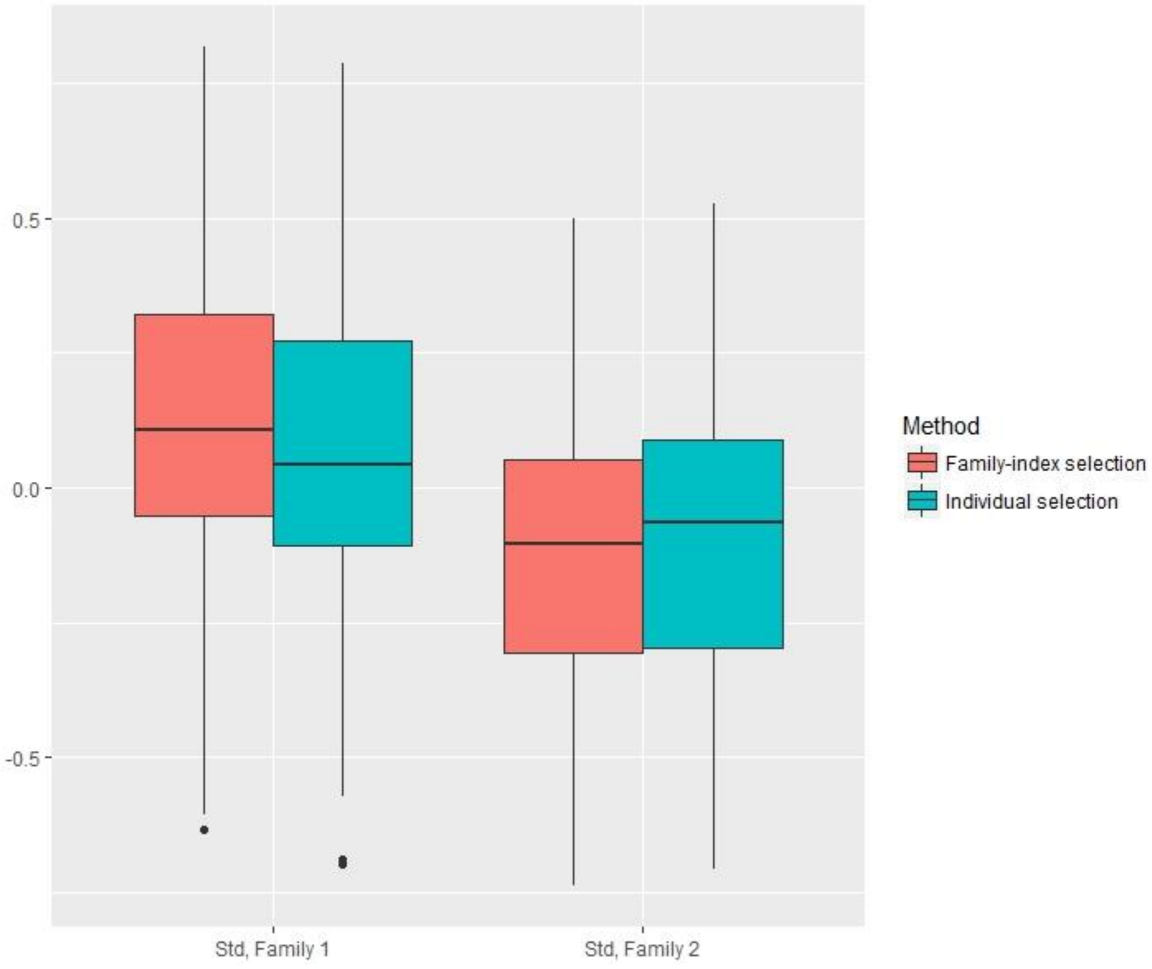

Figure 2A. Standardized BLUPs of flower size in *Dianthus caryophyllus* L., standard carnation

Dianthus caryophyllus L.

Standard carnation

Stem length

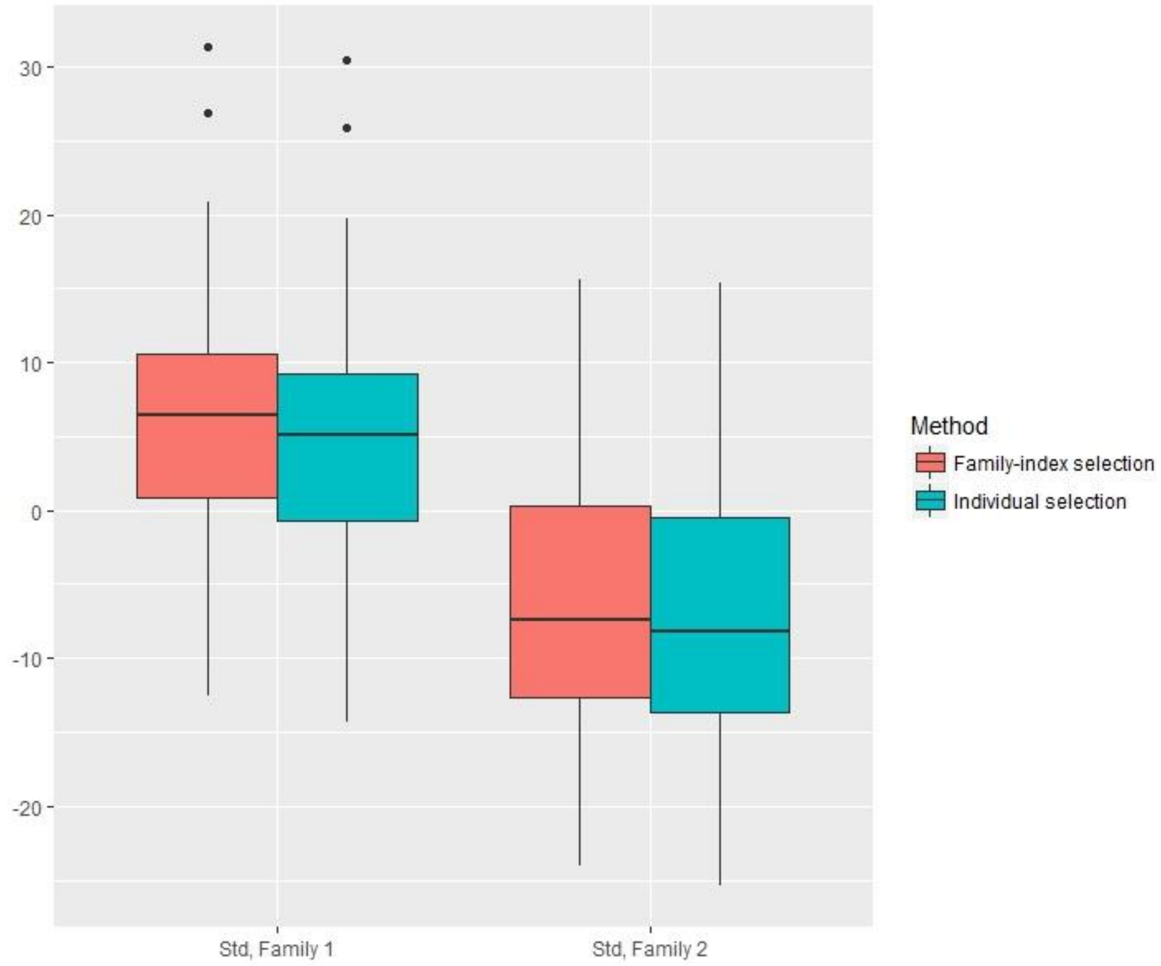

Figure 2B. Standardized BLUPs of stem length in *Dianthus caryophyllus* L., standard carnation

Dianthus caryophyllus L.

Standard carnation

Vase life

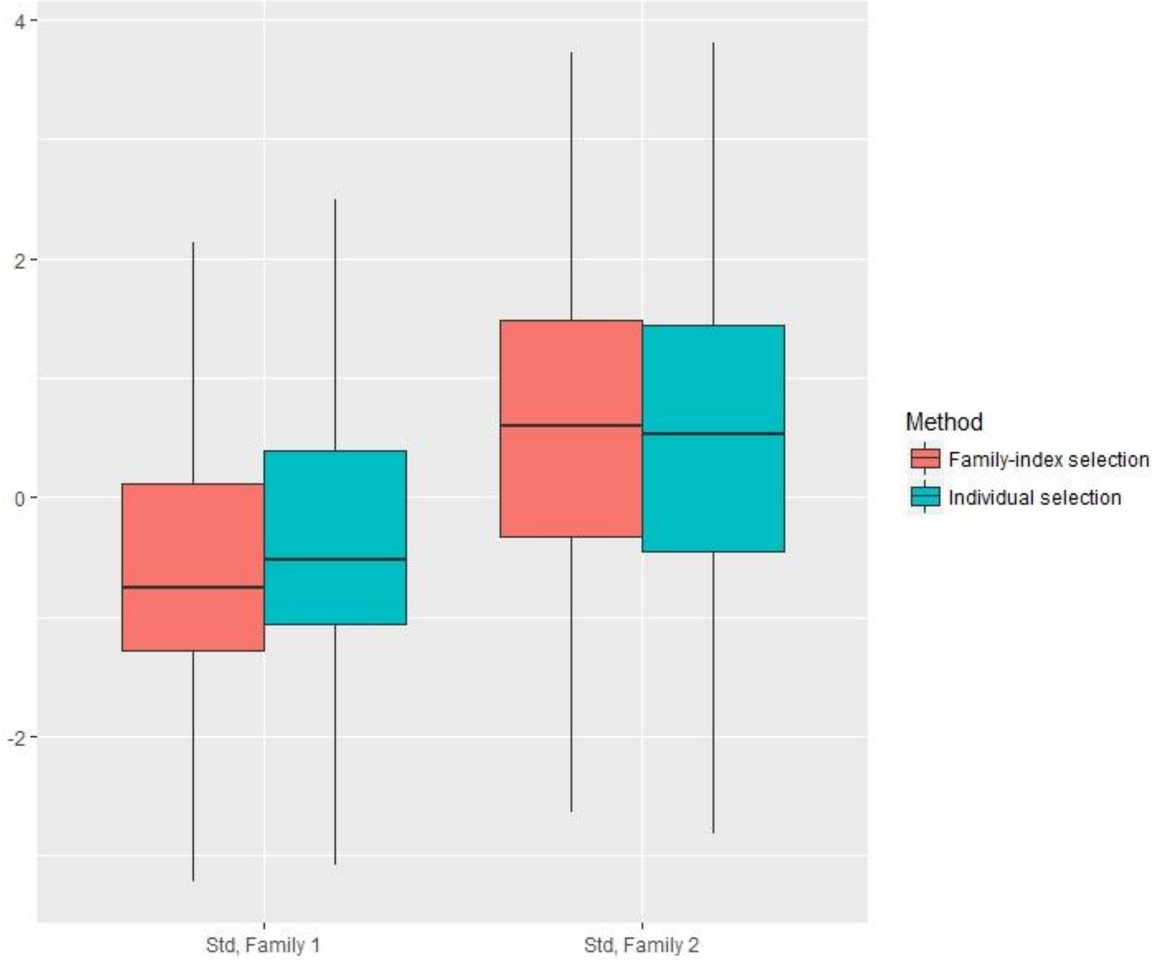

Figure 2C. Standardized BLUPs of vase life in *Dianthus caryophyllus* L., standard carnation

Dianthus caryophyllus L.

Mini carnation

Bud number

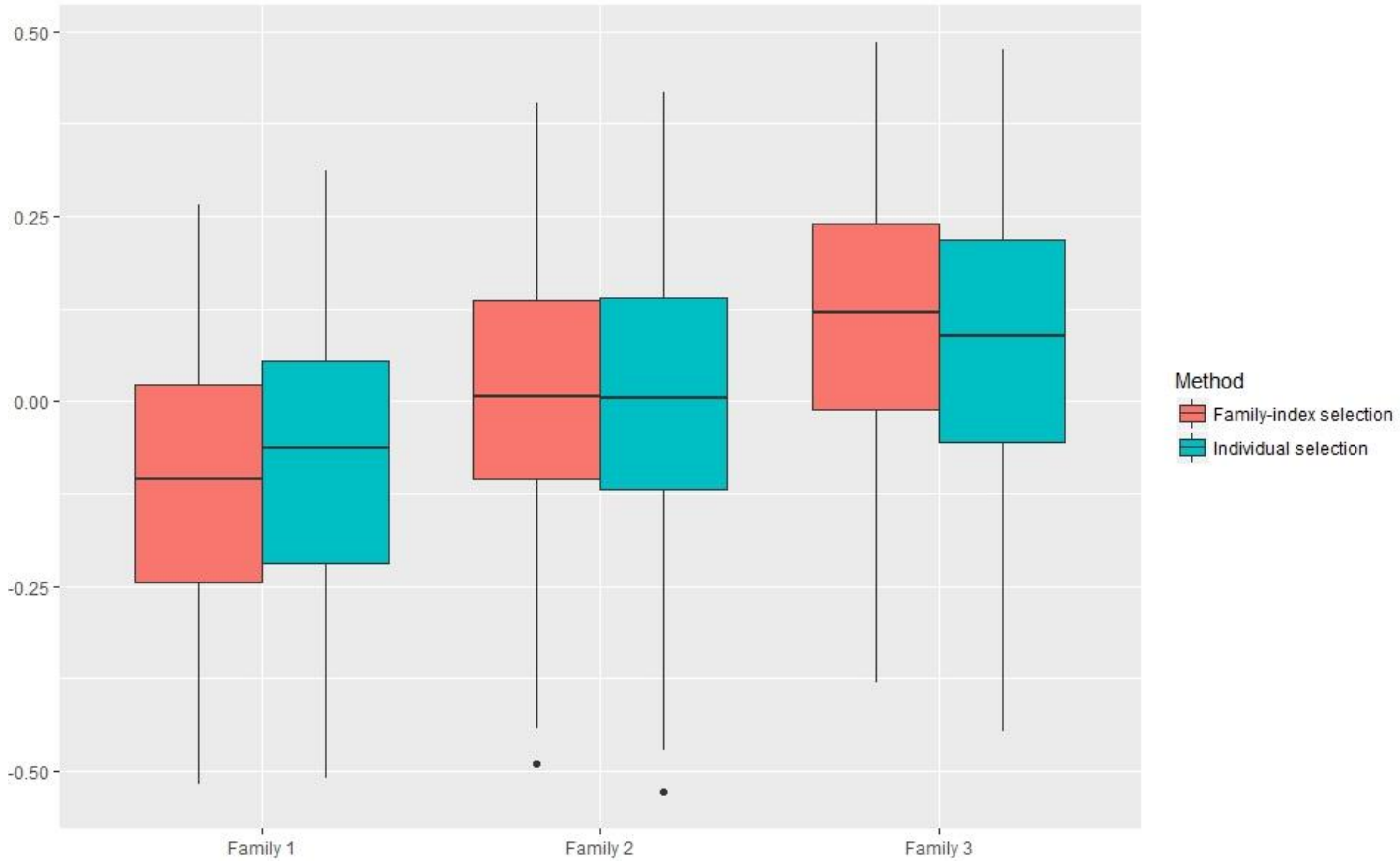

Figure 2D. Standardized BLUPs of bud number in *Dianthus caryophyllus* L., mini carnation

Dianthus caryophyllus L.

Mini carnation

Stem length

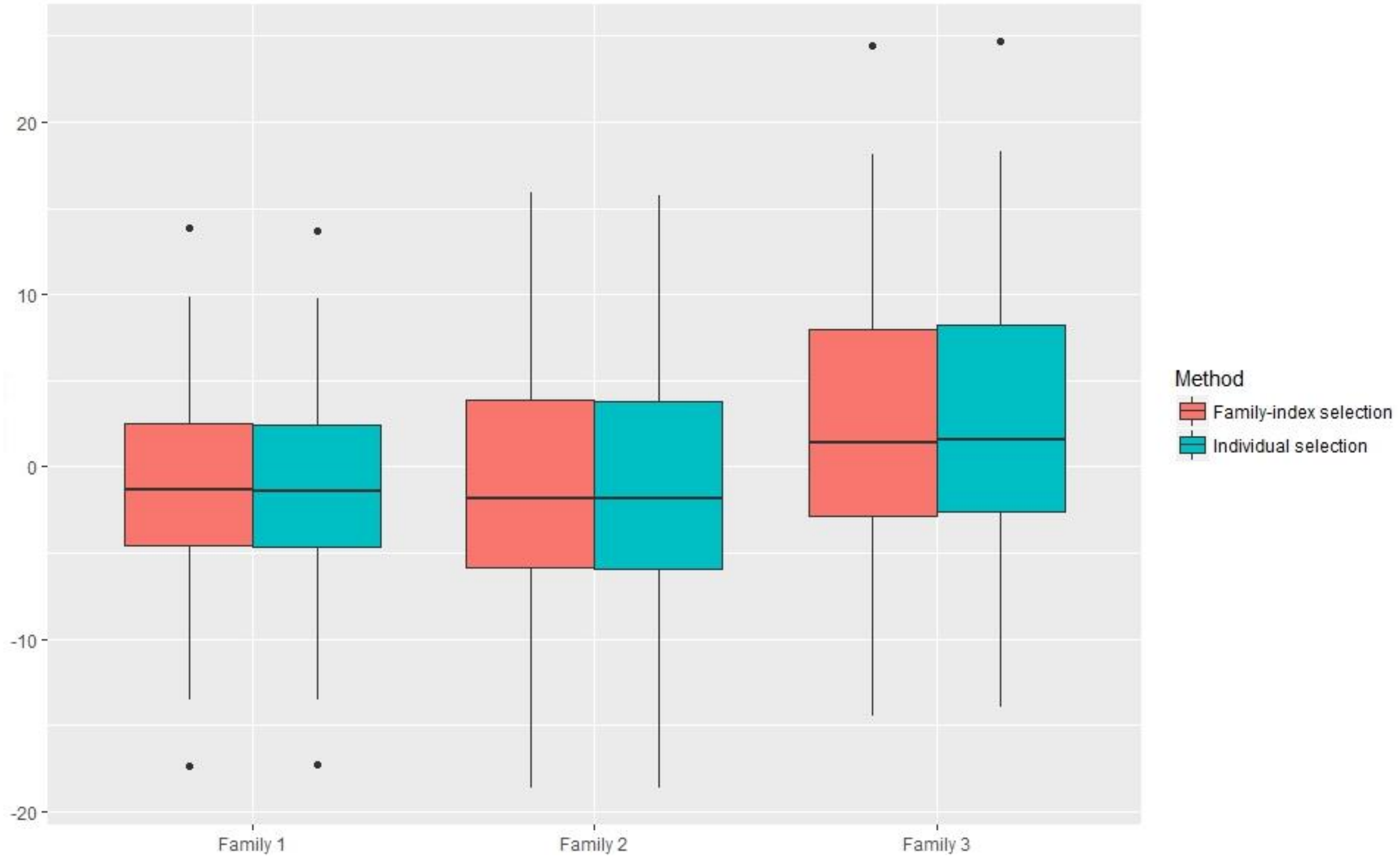

Figure 2E. Standardized BLUPs of stem length in *Dianthus caryophyllus* L., mini carnation

Dianthus caryophyllus L.

Mini carnation

Vase life

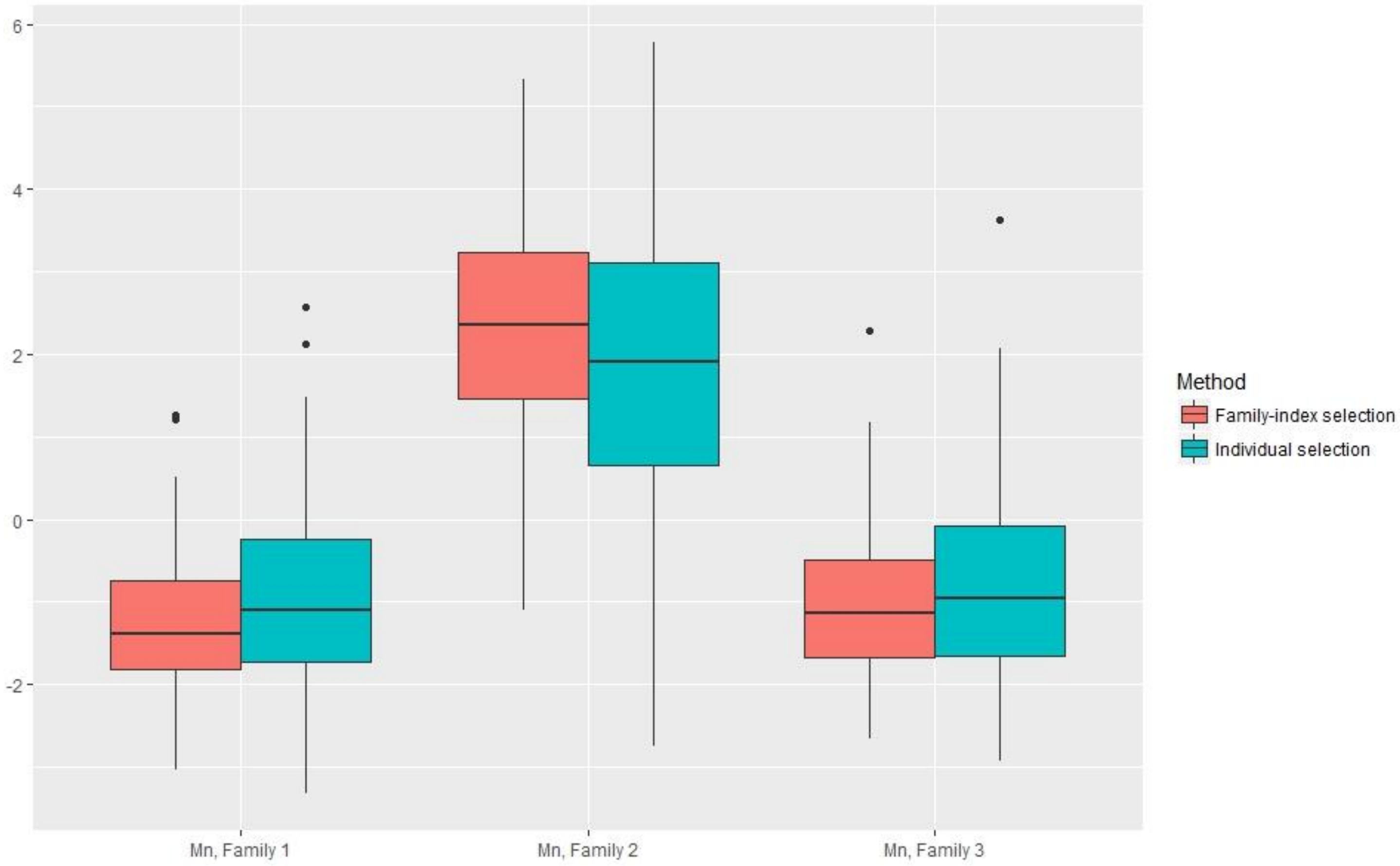

Figure 2F. Standardized BLUPs of vase life in *Dianthus caryophyllus* L., mini carnation

Pelargonium zonale

Stem cutting count

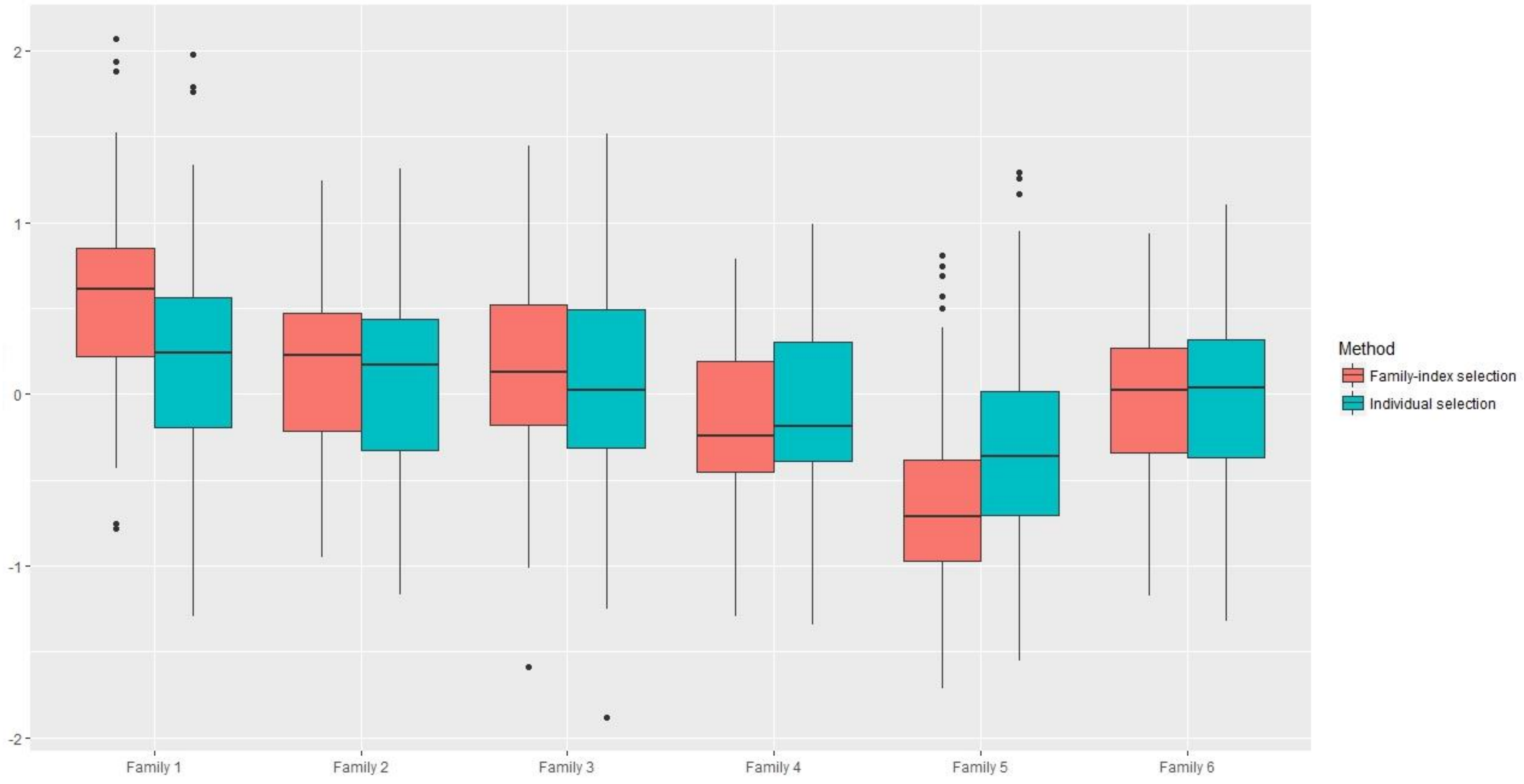

Figure 2G. Standardized BLUPs of stem cutting count in *Pelargonium zonale*

Pelargonium zonale

Root formation

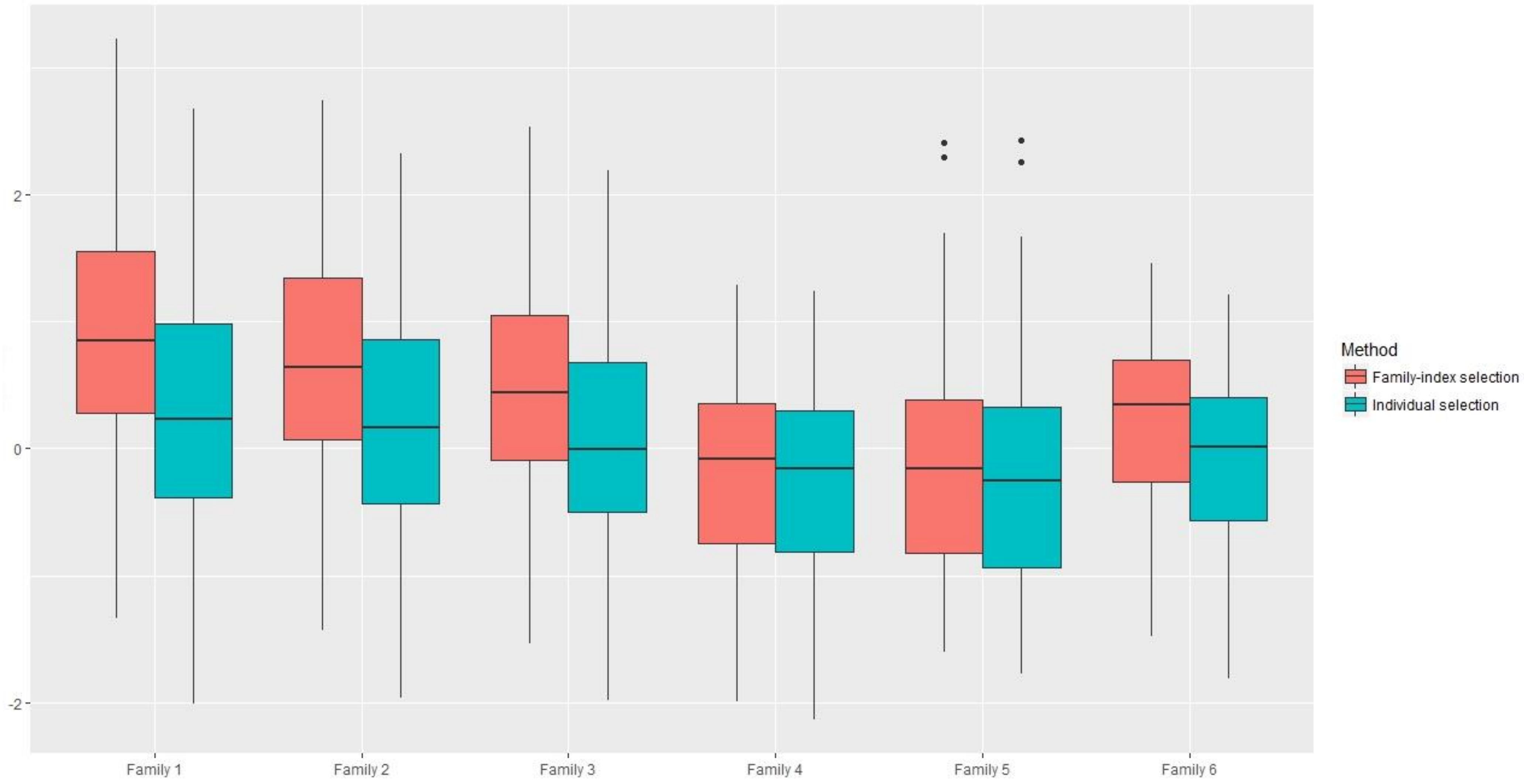

Figure 2H. Standardized BLUPs of root formation in *Pelargonium zonale*
