# Supplementary material for: Phenotypic Selection in Ornamental Breeding: It's Better to Have the BLUPs Than to Have the BLUEs
Source: Front Plant Sci. 2018 Nov 5;9:1511. doi: 10.3389/fpls.2018.01511 (PMC6230591; doi:10.3389/fpls.2018.01511)
Supplement: Supplementary file 3 [file Data_Sheet_3.PDF]

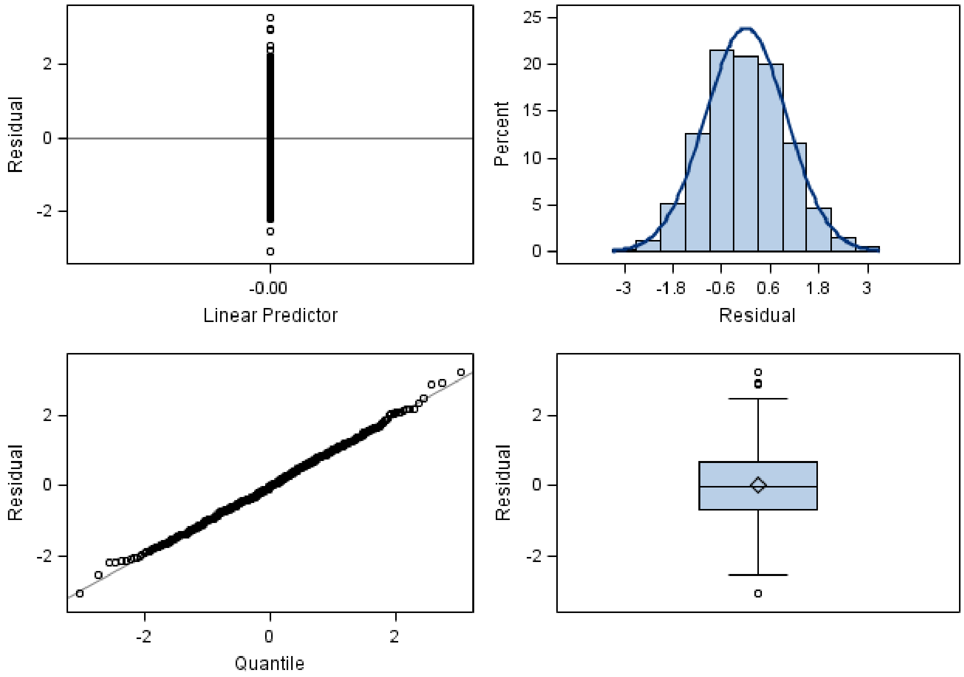

Figure 3A. Standardized BLUP estimates of SCC considering individual selection.

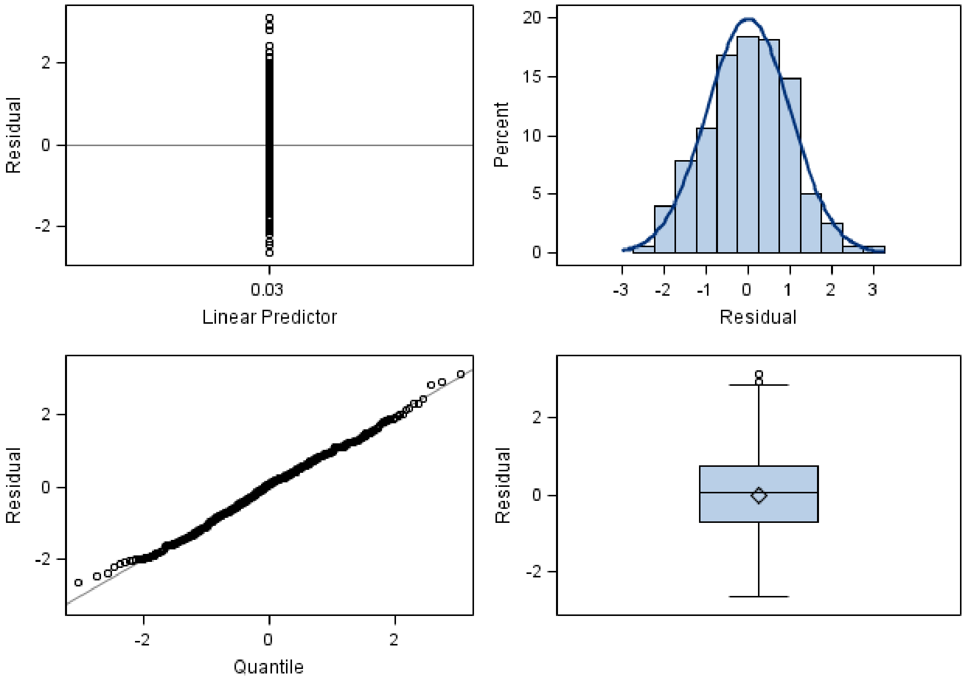

Figure 3B. Standardized BLUP estimates of SCC considering family-index selection.

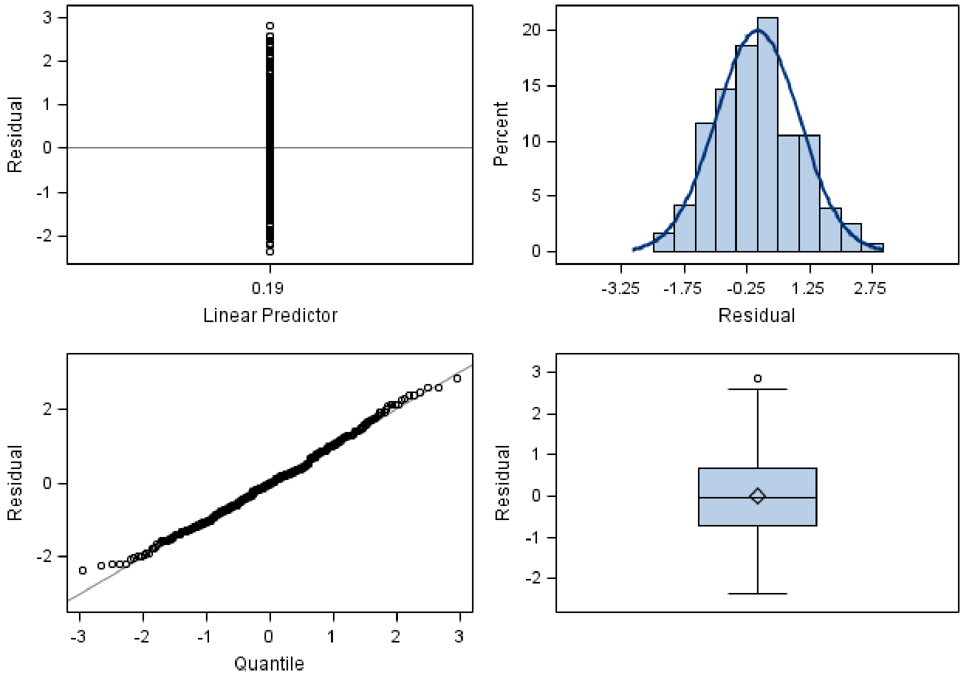

Figure 3C. Standardized BLUP estimates of RF considering individual selection.

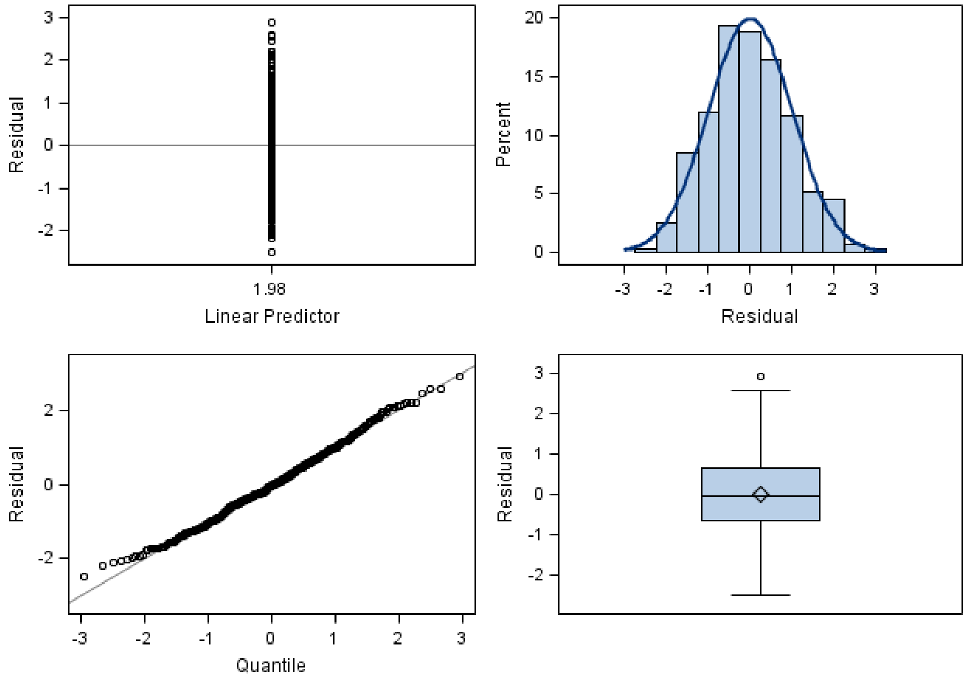

Figure 3D. Standardized BLUP estimates of RF considering family-index selection.

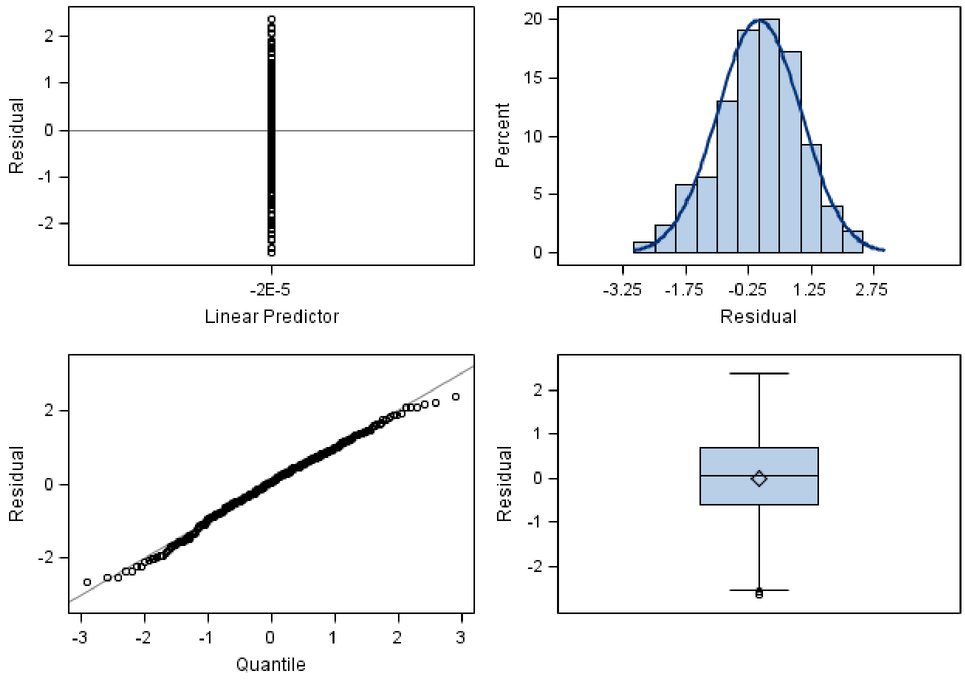

Figure 3E. Standardized BLUP estimates of BN considering individual selection.

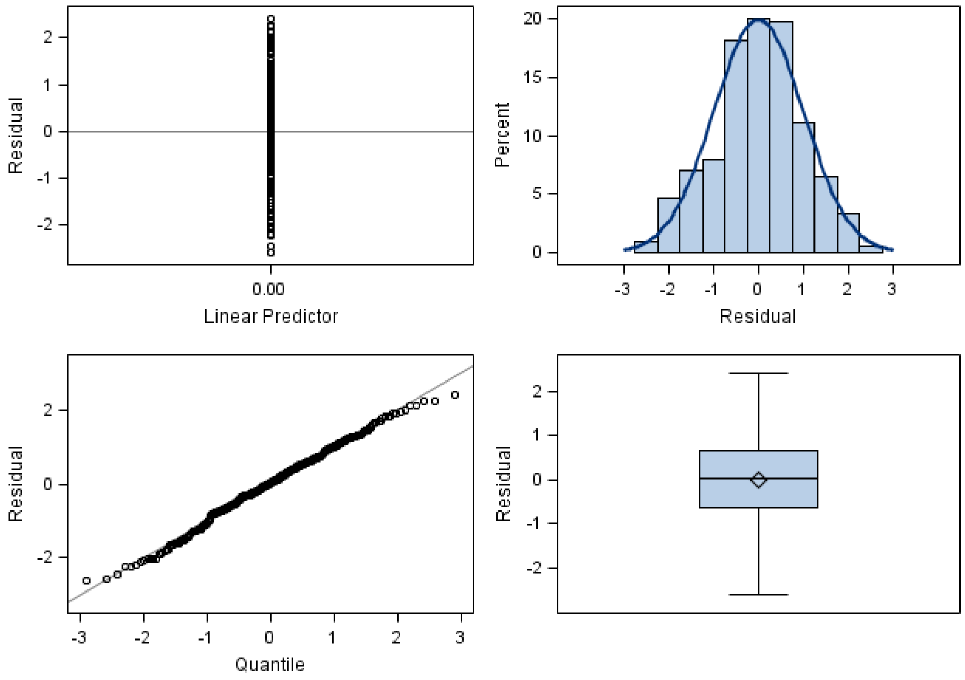

Figure 3F. Standardized BLUP estimates of BN considering family-index selection.

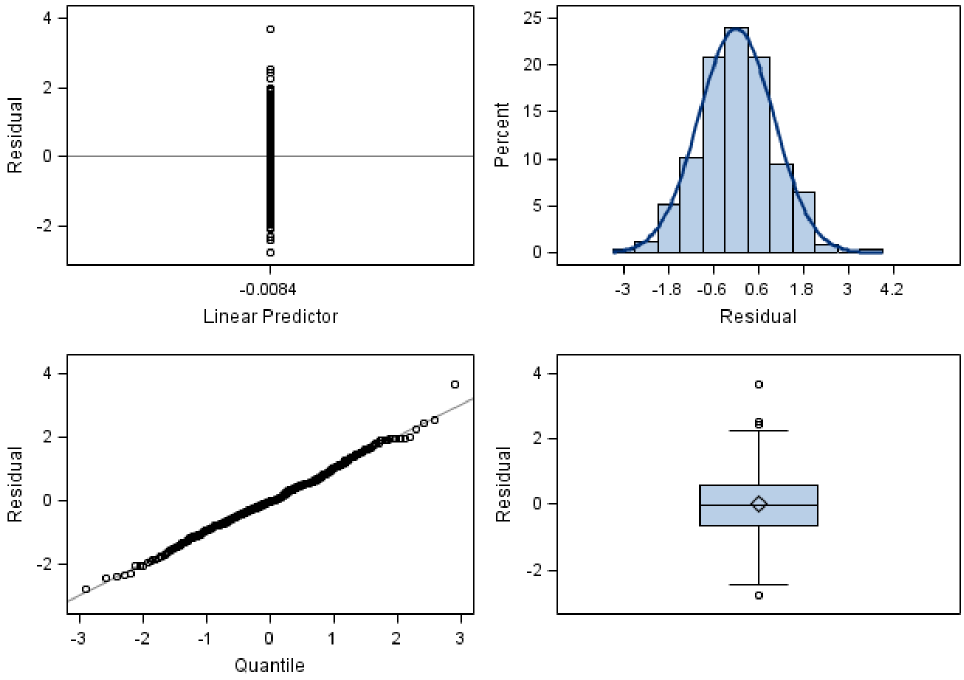

Figure 3G. Standardized BLUP estimates of SL for the mini carnation type considering individual selection.

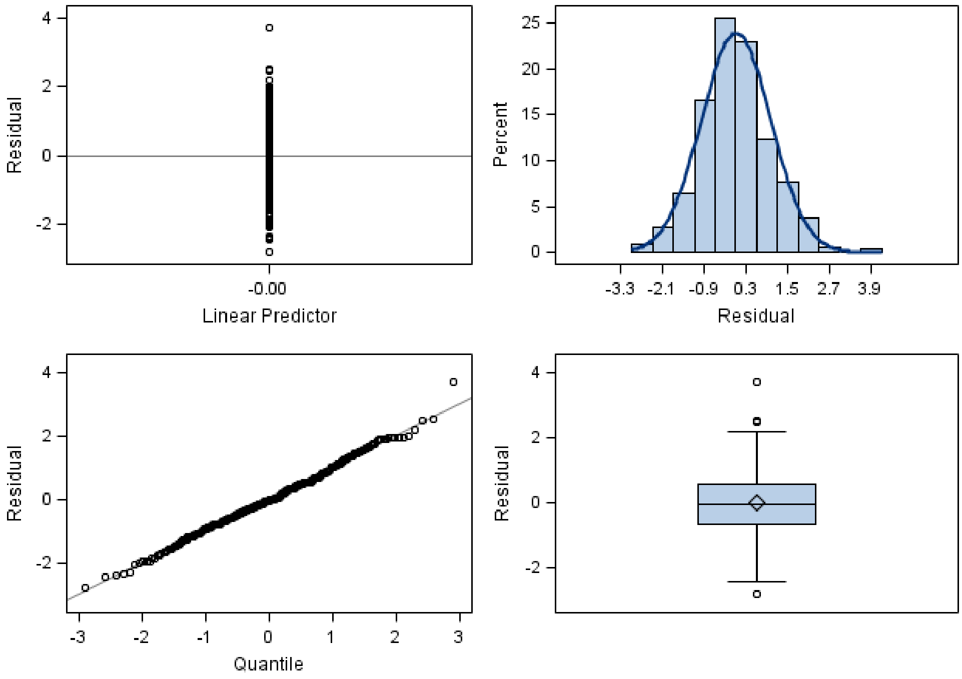

Figure 3H. Standardized BLUP estimates of SL for the mini carnation type considering family-index selection.

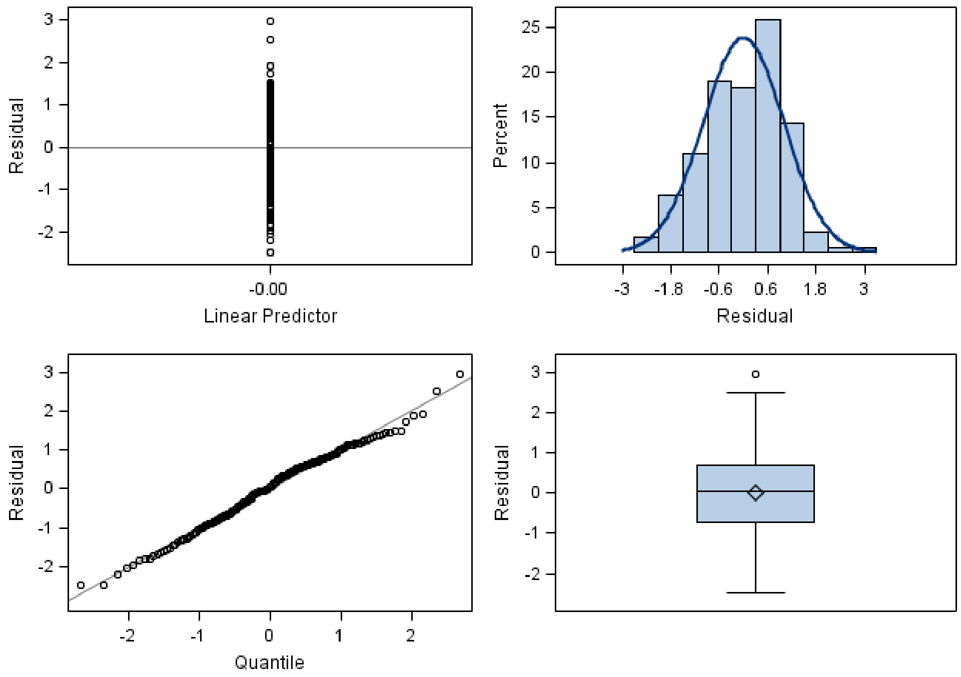

Figure 3I. Standardized BLUP estimates of SL for the standard carnation type considering individual selection.

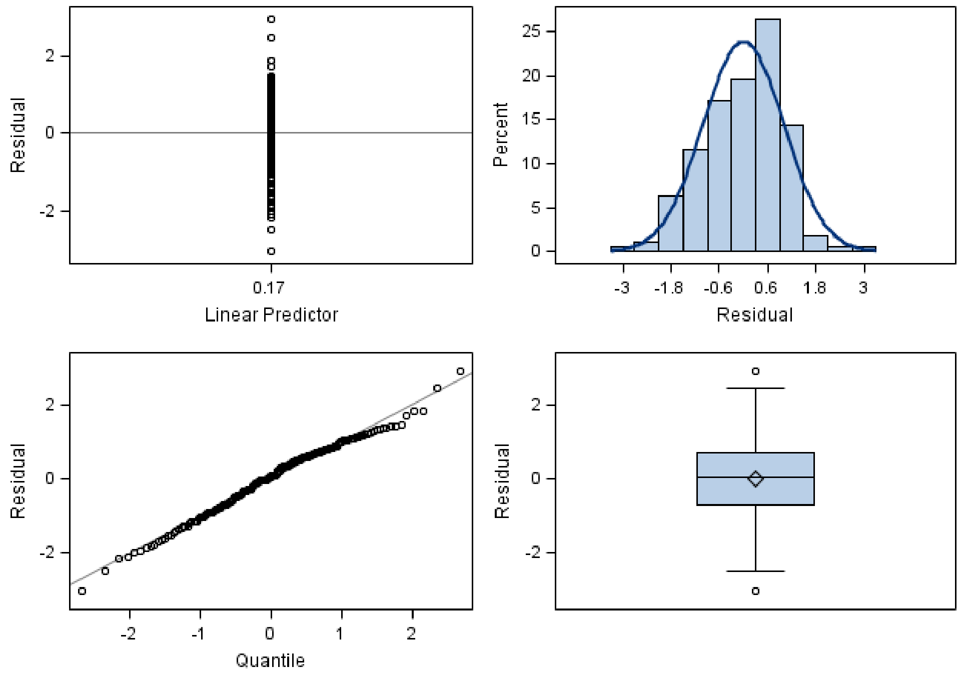

Figure 3J. Standardized BLUP estimates of SL for the standard carnation type considering family-index selection.

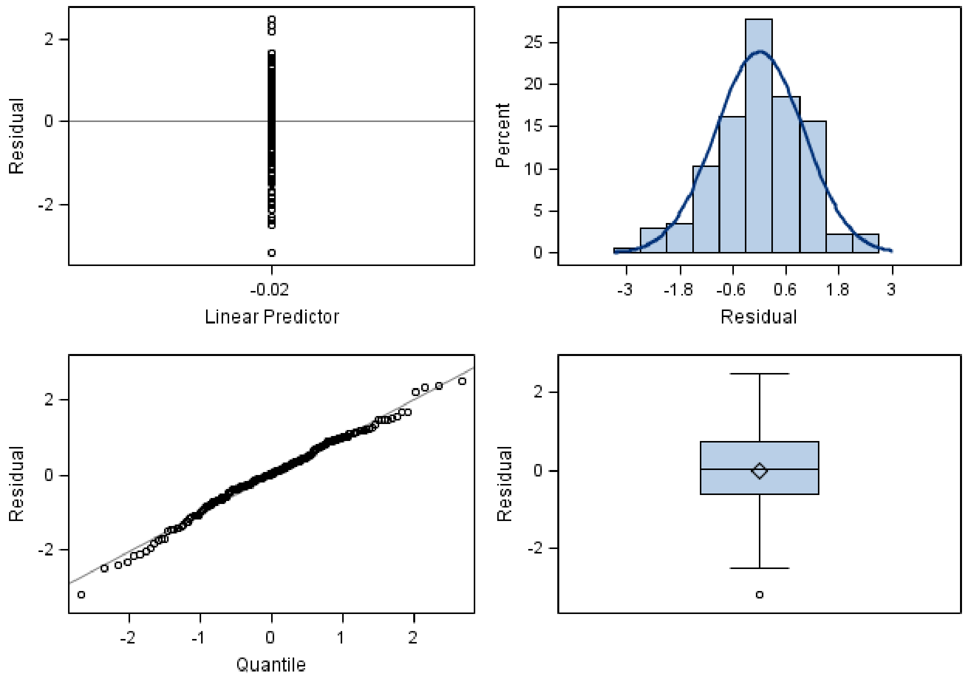

Figure 3K. Standardized BLUP estimates of FS for the standard carnation type considering individual selection.

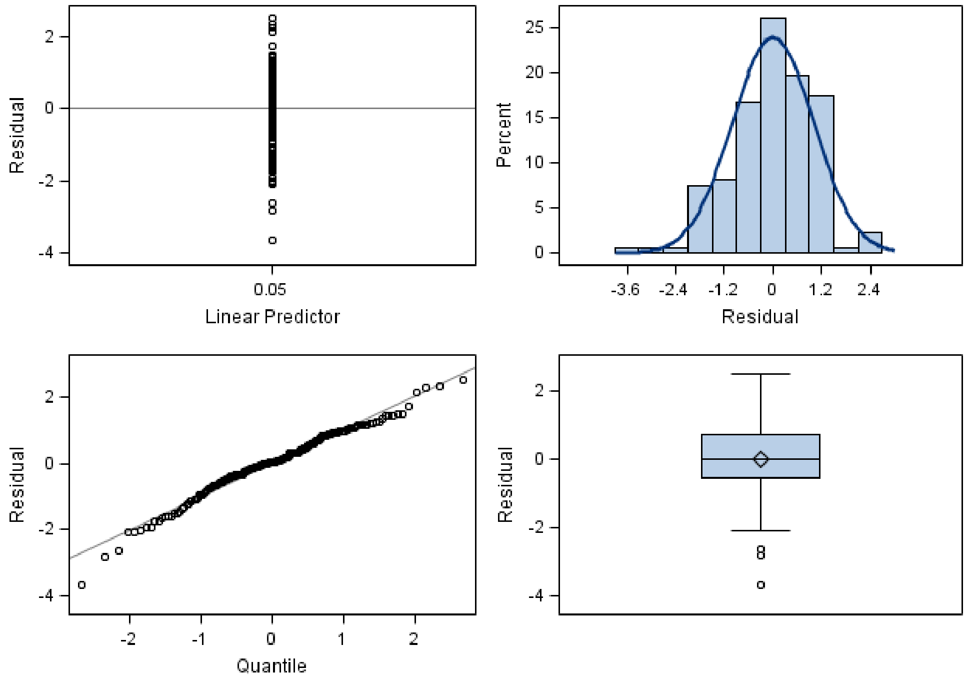

Figure 3L. Standardized BLUP estimates of FS for the standard carnation type considering family-index selection.

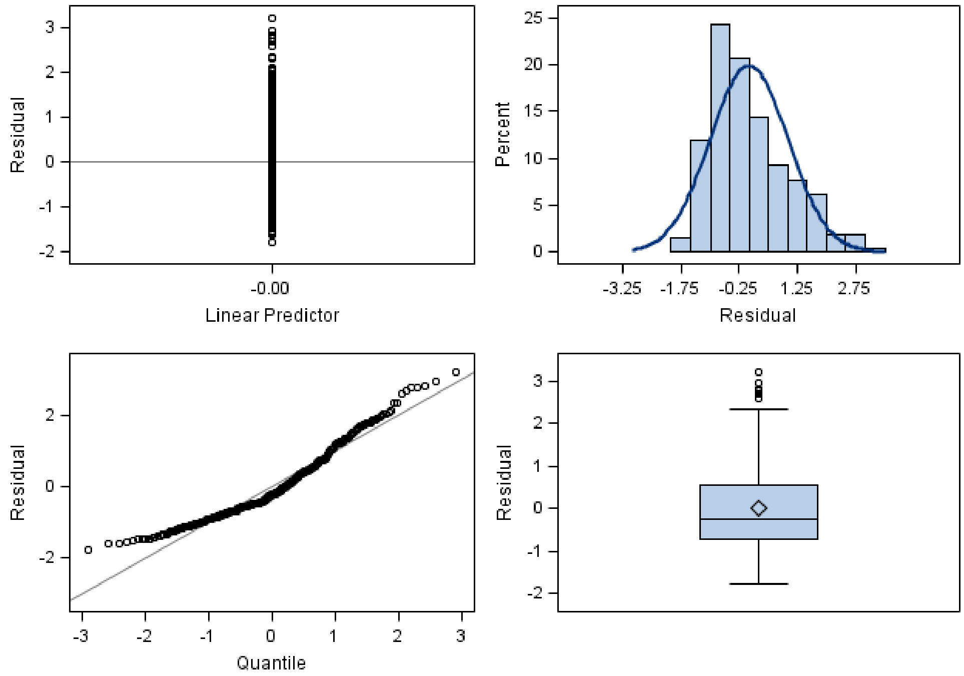

Figure 3M. Standardized BLUP estimates of VL for the mini carnation type considering individual selection.

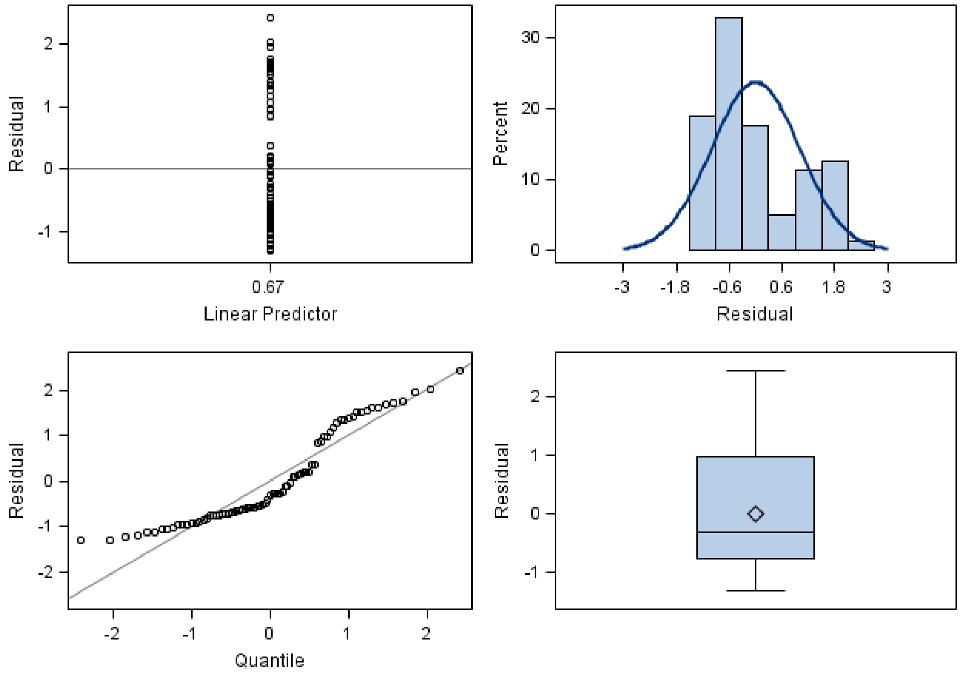

Figure 3N. Standardized BLUP estimates of VL for the mini carnation type considering family-index selection.

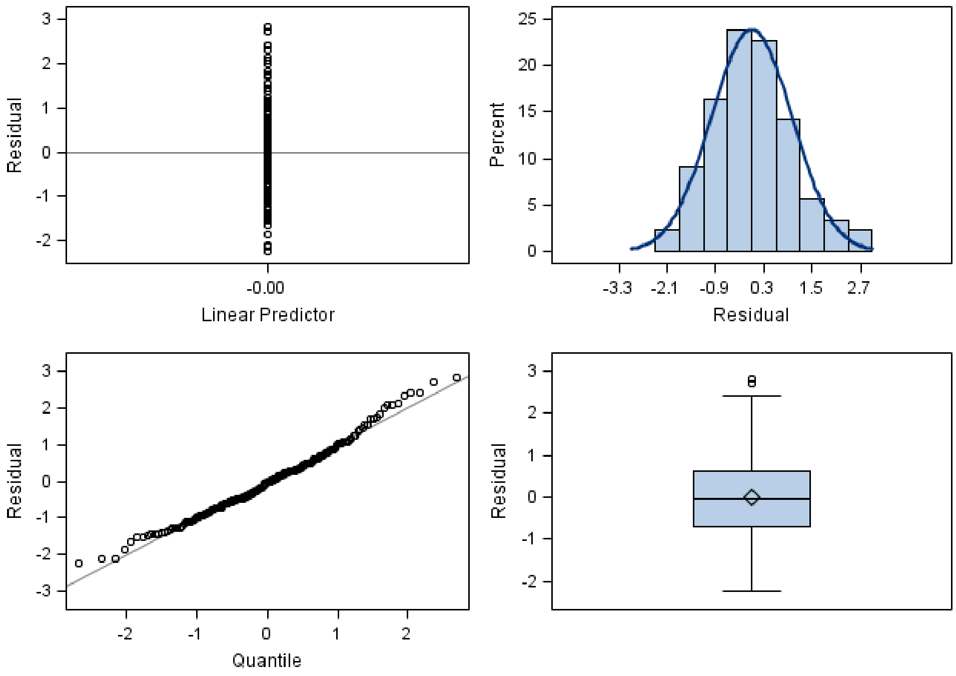

Figure 3O. Standardized BLUP estimates of VL for the standard carnation type considering individual selection.

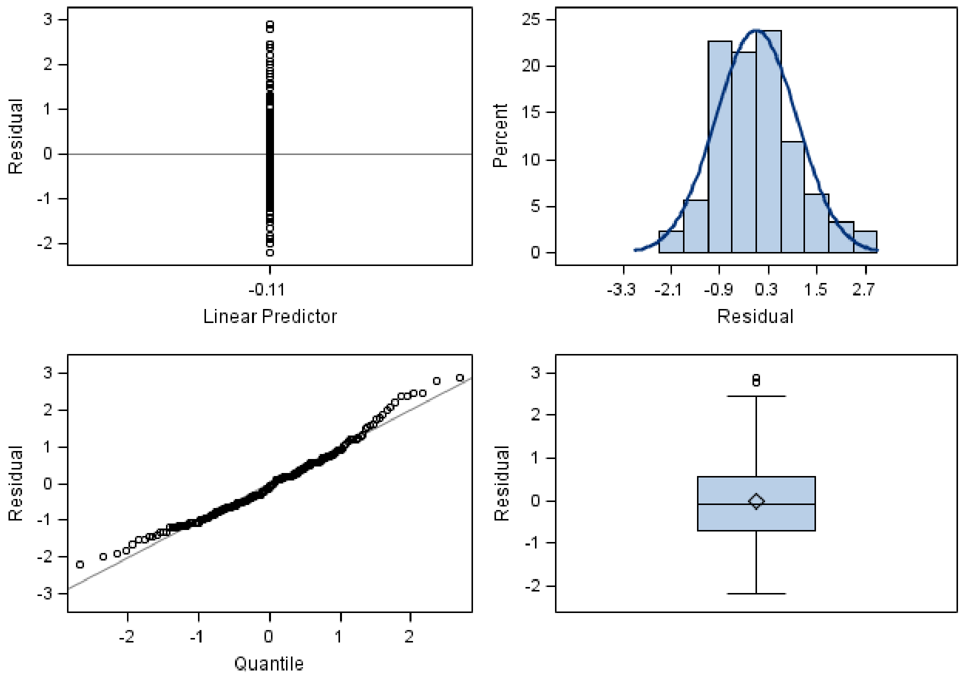

Figure 3P. Standardized BLUP estimates of VL for the standard carnation type considering family-index selection.
